# A Comparison of the Effects of Random and Selective Mass Extinctions on Erosion of Evolutionary History in Communities of Digital Organisms

**DOI:** 10.1371/journal.pone.0037233

**Published:** 2012-05-31

**Authors:** Gabriel Yedid, Jason Stredwick, Charles A. Ofria, Paul-Michael Agapow

**Affiliations:** 1 Department of Biology, Centre for Ecological and Evolutionary Synthesis, University of Oslo, Oslo, Norway; 2 uTest, Southborough, Massachusetts, United States of America; 3 Department of Computer Science and Engineering, Michigan State University, East Lansing, Michigan, United States of America; 3 Centre for Infections, Health Protection Agency, London, United Kingdom; British Columbia Centre for Excellence in HIV/AIDS, Canada

## Abstract

The effect of mass extinctions on phylogenetic diversity and branching history of clades remains poorly understood in paleobiology. We examined the phylogenies of communities of digital organisms undergoing open-ended evolution as we subjected them to instantaneous “pulse” extinctions, choosing survivors at random, and to prolonged “press” extinctions involving a period of low resource availability. We measured age of the phylogenetic root and tree stemminess, and evaluated how branching history of the phylogenetic trees was affected by the extinction treatments. We found that strong random (pulse) and strong selective extinction (press) both left clear long-term signatures in root age distribution and tree stemminess, and eroded deep branching history to a greater degree than did weak extinction and control treatments. The widely-used Pybus-Harvey gamma statistic showed a clear short-term response to extinction and recovery, but differences between treatments diminished over time and did not show a long-term signature. The characteristics of post-extinction phylogenies were often affected as much by the recovery interval as by the extinction episode itself.

## Introduction

The interplay of speciation and extinction is one of the chief mysteries of evolution. Paleontologists have long recognized that life on Earth features a regular turnover of taxa, both from a steady, low rate of species deaths (background extinction) and infrequent but highly destructive episodes that eliminate considerable pre-existing diversity (mass extinctions). Understanding the impact of these is important not only for understanding how they have shaped current biota, but also for projecting future patterns of biodiversity [1]–[6].

In this context, phylogenetic trees are not only a record of the speciation-extinction dynamic, but also a useful gauge for the impact of extinction. Placing taxa within a phylogeny can show the evolutionary distinctiveness of a species (i.e. how long ago it branched from its closest relatives), and thus can be used for assessing the severity of extinction in terms of loss of evolutionary history [4], [7], [8]. However, while mass extinctions have been studied in terms of their removal of diversity and variety as captured by the fossil record, relatively less attention has been paid to their effects on phylogenetic structure (but see [9]–[12]).

Thus, a persistent question has been whether different extinction regimes, e.g. the selective or random "targetting" of taxa, might have different long-term impacts on phylogenies. However, paleontological investigation to this end is thwarted by the unevenness of the fossil record and the difficulty of ascertaining whether any particular episode of extinction from Earth history was causally random or selective [13], [14]. Thus, most literature on this point is either theoretical [15], [16] or in neontological contexts [5], [17], [18], with a few exceptions [19], [20]. One productive approach has been the use of simulated phylogenies generated through branching processes. For example, one study [16] modeled extinction that was random or selective with respect to a trait controlling speciation rate, finding that each extinction type had different effects on tree balance: random extinction produced more imbalanced trees, while the opposite was true for selective extinction. However, while this simulation approach excels in analytical tractability, it inevitably simplifies and abstracts the complexities of real-world ecologies, studying phylogenies that grow and die in isolation from any other taxa.

In a previous study [21], we used a digital ecosystem to compare the effects of different extinction regimes. In this way, we were able to capture the advantages of both simulation and paleontological approaches and showed that when comparing random and selective extinction –as implemented in pulse (an instantaneous mass random) and press (environmental restriction) events– ecologies affected by press events required greater recovery times. Here we consider these different regimes in phylogenetic terms and ask i) whether they differ in terms of impact on the phylogenetic structure of communities, and ii) whether *post facto* such regimes leave, and can be distinguished by, any permanent phylogenetic signature.

## Methods

### The Digital Ecology

As opposed to previous work on extinction patterns – which used phylogenies either reconstructed from paleontological data or those simulated in branching model studies [4], [5], [8], [11], [12], [15], [16], [22], [23] – here we study extinction patterns derived from an ecology of digital organisms, implemented in the artificial life system Avida (v2.1, available from avida.devosoft.org) [24]. Avida is a digital ecosystem that features variation through mutation, vertical inheritance of evolved characteristics, and competition for limited resources: the three major ingredients necessary for Darwinian evolution. It has been used to examine questions as diverse as mutational robustness and evolvability, evolution of complex, multi-part features from simpler precursors, emergence of stable ecosystems through density-dependent selection, and more [25]–[34]. However, it has not yet been applied to investigating effects of evolutionary processes on phylogenetic tree shape. This artificial life approach can be seen as one of a variety of complementary approaches for modeling modes of recovery and extinction including trophic network models [35], paleocommunity food web models [36], [37], [38] and analytical models [39]. Avida’s unique adavantages are that it is individual-based, and features evolutionary effects based at the genomic level and emergent behaviours largely lacking in these other classes of models. Modeling in turn is complementary to empirical and paleontological approaches, but instead of trying to understand observed patterns forensically [40], [41], attempts to find mechanisms that produce these patterns.

Avida has been detailed at length elsewhere [24] as has our experimental setup [21], [29], but some of the details of the implementation are salient. First, the ecosystem has a limited amount of space, introducing an element of drift as new organisms displace the old. Second, the environment features multiple depletable resources, linked to certain computations, where a low concentration of a resource reduces the benefit gained by performing an associated computation. This feature mimics density-dependent competition for resources, favouring organisms that most efficiently consume particular resources or target under-utilized ones. Resources are globally available to all organisms, with no spatial structure. Only a limited number of resources are supplied exogenously, while the remainder can only be generated as “by-products” by organisms when they successfully complete certain associated computational functions (these interactions are discussed at greater length in Data S1, and illustrated in Figure S1). This introduces ecological interdependence into the population dynamics, which is a feature of real communities. Configuration files for performing the experiments described here are available in File S1.

As stated, this digital approach combines a number of advantages over the previous paleontological and branching model studies. As compared to paleontological studies, experimenters have omniscient knowledge and control of the system, rather than being forced to find and interpret appropriate scenarios. In turn, this allows the easy production of replicates for statistical analysis. Conversely, phylogenetic and evolutionary processes are not abstractly modeled as in branching models, but are actual emergent dynamics of the system that can be directly analyzed. Extinction selectivity is mediated through changes in the environment, not by directly selecting tips of a phylogeny. This approach is more complex but more realistic: extinction is not an ecologically isolated event but can have consequences and knock-on effects beyond the targeted clade. This is in contrast to branching process models, where speciation and extinction occur in isolation, with no effect or response from the outside world.

Some caution must be expressed about the interpretation of Avida dynamics into phylogenetic patterns. Like a colony of asexual clonal bacteria, each organism could be seen as its own lineage and classifications such as “species” must be defined *post hoc* [30], [31]. As genotypic differences are the clearest distinction, we use them as the basis for cladogenesis. A cladogenetic event occurs when an organism gives rise to two or more organisms with genotypes that differ from its own. Each is then an independently evolving lineage–implicitly an evolutionary species concept [42]. Note that this does not imply that the offspring differ in phenotype. Cladogenesis occurs largely through production of near-neutral variants of a parent genotype, decoupling branching from anagenetic change and resulting in a tendency towards trees with long basal branches. This topological bias is exacerbated by several factors, including the high mutation rates and finite population sizes common to Avida experiments, but also diversifying selection for adaptation to different sets of resources [43]. In model systems where phenotypic change is more tightly coupled to speciation (so-called “punctuational” systems [44]), trees tend to be less “stemmy” but it is difficult to argue which mode of cladogenesis is more or exclusively realistic. Varying mutation rates thus increases or decreases the number of branching events per unit of absolute time, also affecting total tree size. Further, while the organisms are completely asexual, we note that the branching process models usually used to study tree shape make no assumptions about mode of reproduction; asexual organisms can form phenotypically distinct phylogenetic clusters just as well as sexual organisms [43]. Recombination would also result in reticulate (as opposed to tree-like) phylogenies.

### Experimental Methodology

We examined and compared random and selective extinction regimes in terms of “pulse” and “press” extinctions. In macroevolutionary terms, a pulse extinction happens with sufficient speed that there is insufficient time for adaptive change to occur during the episode, although adaptation may take place afterwards. This is implemented in the digital ecosystem as an instantaneous mass culling of individuals from the population, with survivors selected at random from among those organisms capable of self-replication. By contrast, a press extinction occurs over a longer period that allows for an adaptive response in affected populations [45]. This is implemented as a period of altered environmental conditions–greatly reduced inflows of basal resources–that persists long enough to allow an adaptive response. Ecological recovery is then initiated by restoring resource inflows to pre-extinction levels. This presents a more plausible scenario for extinction selectivity than simple targeting of specific traits as in branching models. An ideal framework for modeling extinction events would be able to capture independently adaptation during the event, the degree of kill and selectivity of kill. In reality, and certainly in Avida, severity and duration are not independent of each other in the case of a press extinction – a longer press episode may lead to more extinction. Further, selectivity is an emergent aspect of the system and not explicitly set. There are many criteria (phenotypic traits, clade membership, etc.) on which selectivity may be based in the case of a pulse extinction; it is difficult to justify any one over another.

The following experimental treatments were applied here; the first three are from [21]:

1. Uninterrupted evolution (control). Each replicate ran unperturbed for 205,000 updates.
2. Strong press episode. Each replicate ran for 100,000 updates. Resource inflows were then lowered by two orders of magnitude for 5,000 updates. This treatment was applied uniformly across experiments as absolute time, much as real extinction-driving crises act independently of the generation times of the organisms affected. Resource inflows were then restored for a subsequent 100,000 updates, yielding equal amounts of absolute time for pre-extinction and post-extinction evolution.
3. Strong pulse extinction. Pre-extinction histories were as before. At 100,000 updates, an instantaneous mass cull of the population was performed. Survivors were picked randomly from among viable organisms, with no environmental alteration. This treatment was followed by 100,000 updates of recovery, again equal to the time for pre-extinction evolution, though the total length of the experiment was shorter. The survival rate of this strong cull is 4/3600 individuals (0.1%). Although this may seem extreme, such levels can occur (e.g. ammonoids at the end-Permian extinction [46]).
4. Weak press extinction. Pre-extinction histories and press duration were as before, but the reduction in resources was not as severe. The low resource level was about 10-fold higher than for the strong press; this setting most closely matched the phylogenetic attrition of the weak pulse treatment (summary data in Table S1).
5. Weak pulse extinction. Pre-extinction histories were as before, but the survival rate of the cull was 36/3600 individuals (1%).

Transformation of Avida population data into phylogenetic trees was accomplished with software routines custom-written by JS (the software is available in File S2). The phylogenies thus produced were “molecular”, since the tips were only genotypes extant at the time of sampling. These routines also produced lineage-through-time (LTT) data for each tree, as well as calculating tree shape metrics (see below). Node ages represented branching times for ancestral genotypes that had at least two lines of descent surviving until the time of sampling.

### Data Analysis

The effect of these different extinction treatments on tree structure was examined as follows:

#### 1. Deep vs shallow structure

Our previous work [21] demonstrated that the altered ecological conditions of the press episode strongly favoured organisms with short generation times and low investment in ecological functionality, able to subsist mostly on a basal resource provided to all organisms that enables any execution at all. Such organisms came to dominate the population, like paleontological “disaster taxa.” This dynamic did not produce the same obvious loss of “biomass” inherent to a pulse extinction, so that many lines of descent might survive the press episode through decay into simple replicators. Did the press episode, then, produce genuine removal of deep phylogenetic history?

We used LTT data produced for calculation of the Pybus-Harvey gamma statistic (see below) for the pre-extinction and post-event trees, with all nodes originating after the beginning of the extinction event discarded. (Loss of a branching node from LTT data is equivalent to loss of all but a single descendent lineage of the genotype that node represents.) For each individual phylogeny, we binned the node ages from the LTT data temporally into intervals of 5000 updates. For each replicate, nodes in each bin were counted for the population immediately before the extinction, at the end of the press episode, the corresponding time period from the control, and in post-pulse trees. For each temporal bin, we calculated the percentage of nodes that persisted by comparing the number of nodes in that bin at the later time point to the number in the pre-extinction population’s corresponding bin. Bins that were empty at both time points were excluded from analysis. An average percent persistence was calculated over the whole data set for each bin. Scores of zero were included in the averages since this represented loss of all branching events that occurred in that bin.

A worked example of the procedure is shown in Data S2 (Figure S4).

#### 2. Stemminess

As different tree topology metrics respond to different aspects of a given tree, two algorithmically distinct measures were used to assess tree stemminess:

- the gamma statistic of Pybus and Harvey ([47], here abbreviated **PHG**). This widely used statistic measures diversification from using internodal distances with negative values indicating nodes are concentrated towards the root, which is usually interpreted as a decrease in diversification over time [48]. For calculation of PHG at various time points, the “present” was taken to be the time of sampling.
- the noncumulative stemminess of Rohlf et al. ([49], here abbreviated **NCS**): This is measured using the actual branch lengths within the phylogeny. NCS values less than 1 indicate long branches from the root before the next branching events; values greater than 1 indicate short branches near the root.

The mathematical definitions of these metrics are given in Data S3Data S3.

For all replicates, we calculated PHG and NCS for the immediate pre-extinction population, the immediate post-extinction population, and the following time points into the recovery: 2000, 5000, 10000, 25000, 50000, 75000, and 100000 updates.

### Statistical Analysis

We employed one-way ANOVA with Tukey-corrected multiple comparisons at each time point to compare differences in both percentage retention of nodes and number of nodes in temporally-binned LTT data, and PHG values among experimental treatments. For NCS, we employed a similar procedure using nonparametric Kruskal-Wallis tests due to extreme non-normality of NCS values both within and among treatments. All analyses were performed using the Statistics Toolbox in MATLAB R2010b [50].

**Figure 1:**
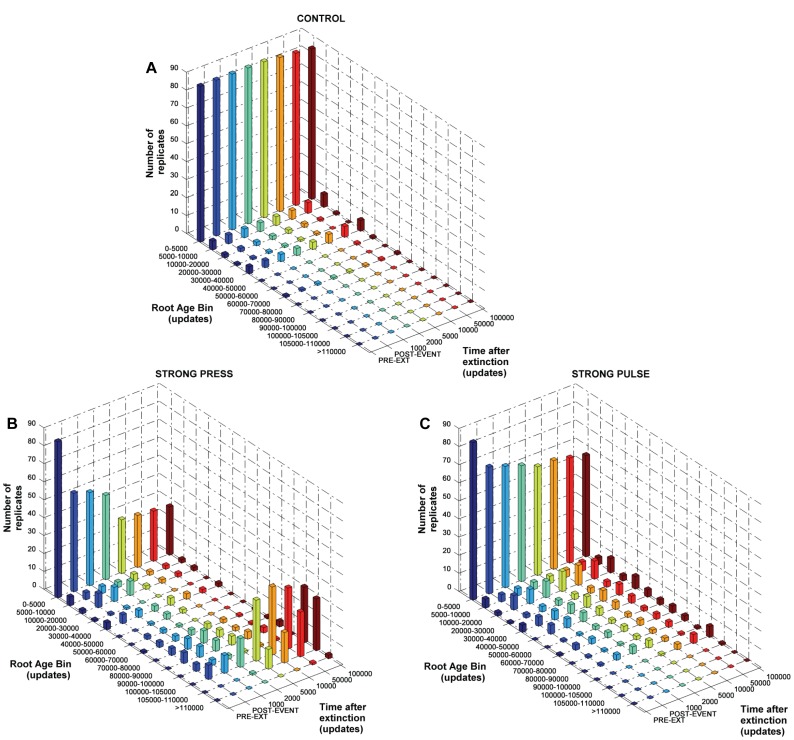
Shift in the distribution of phylogenetic root ages for a) control, b) strong press and c) strong pulse extinctions. Coloured bars show number of replicates (y-axis) with phylogenetic roots whose time of origin falls into the specified root age bins (x-axis). Distributions of root ages were recorded at the time points shown along the “time after extinction” axis (z-axis). Results for Weak press and Weak pulse (not shown) are similar to panel a).

For statistical comparisons involving PHG, only those replicate populations that maintained the pre-extinction phylogenetic root to the end of the experiment were analyzed. This was done so as to avoid comparisons between trees with very different heights (distances from root to point of sampling), as Strong Press trees in particular often ended up with much later-originating roots after the extinction and recovery (see Results, Figure 1). As a result, the number of included replicates declined over time, particularly for the Strong treatments. The results for NCS differed depending on retention of the root, so those are treated separately.

## Results

### Retention of Pre-extinction Phylogenetic Root

The extinction regimes differed in their effect on the age of the phylogenetic root, i.e. the population’s most recent common ancestor at sampling time (Figure 1). Just before the extinction event, 87% of replicates featured a root originating within the first 5000 updates of the experiment; only one replicate’s root originated after 50,000 updates. Only 11% of controls lost the pre-extinction root by the end of the experiment, indicating loss of an entire subclade that branched from the previous root. However, most roots still originated before 50,000 updates (Figure 1a).

Strong Press produced a large shift in the distribution of root origins. Only 52% of replicates retained the pre-extinction root through the press episode, and the distribution of origin times became spread out along the pre-extinction period (Figure 1b). Of note, eight replicates lost all pre-extinction branching, i.e. all diversification at the end of the experimental period originated during or post-extinction. The early recovery continued to reduce the mode of early-originating roots, indicating further loss of deep-branching clades post-extinction. By the end of the experiment, 27% of replicates featured a root older than 5000 updates, and about as many with a root originating either during (31%) or even after (29%) the press episode (Figure 1b).

Strong Pulse also produced a substantial shift in the distribution of root origin times, though not as drastic as Strong Press (Figure 1c). The post-extinction recovery continued to reduce the pre-extinction mode of old roots and resulted in a long tail of later-originating roots, resembling the end of the Strong Press episode. By the end of Strong Pulse experiments, only 48% of replicate populations retained the pre-extinction root, with 56% of replicates in total featuring a root older than 5000 updates. In contrast to Strong Press, only one replicate’s root originated following the extinction. The weak treatments had very minor effects on the distribution of times of root origin, with results similar to those of Control (not shown).

### Loss of Phylogenetic Tree Structure and History

**Figure 2:**
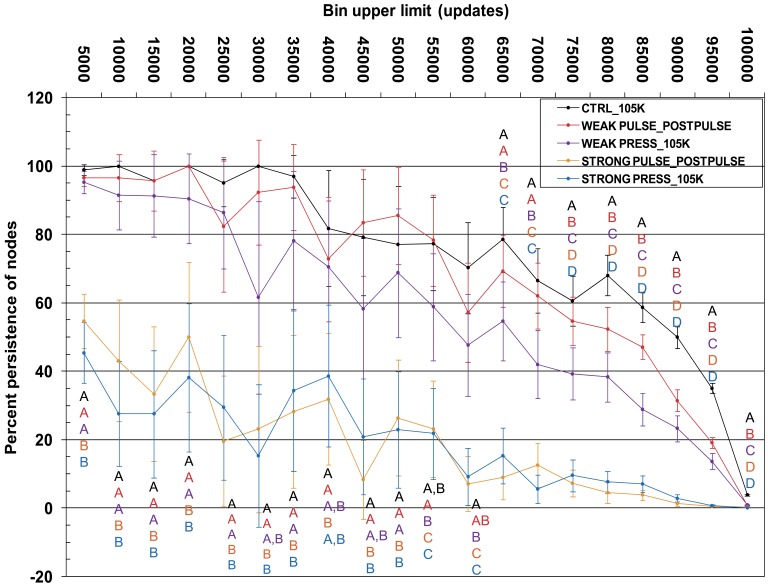
Plot of percent retention of nodes for temporally binned lineage-though-time (LTT) data. Temporal binning was performed as described in Methods (also see example in Data S1), using bin sizes of 5000 updates. The y-axis shows percent retention; the x-axis is time, divided into bin categories. The listed ticks show the upper limit of each bin. Percent retention is calculated for each replicate, relative to the number of nodes that were present in the bin at the immediate pre-extinction time point (t = 100,000 updates). Data for each bin are averages across only replicates where the bin contained at least one node in the pre-extinction LTT data (see Methods). Error bars are two standard errors. Differences between treatments were tested with one-way ANOVA followed by Tukey-corrected multiple comparison tests. Letters indicate treatments that are NOT significantly different from each other after correction for multiple comparisons, and are colour-coded by treatment.

In control experiments, over the period of absolute time corresponding to the press episode (100,000–105,000 updates), background extinction affected branching events nearest the extinction horizon most strongly (Figure 2, black trace). In bins older than 40,000 updates, retention of nodes averaged between 95.1% ±6.86 and 100%, indicating that the deepest parts of the pre-extinction phylogenies remained mostly intact. This was also true for Weak Pulse (Figure 2, red trace), which differed significantly from Control only in bins from 75,000–80,000 updates onwards. Retention was consistently lower in bins beginning at 60,000–65,000 updates, but did not differ significantly from the control. Retention in Weak Press was significantly lower than Control and Weak Pulse from 65,000–70,000 updates to the extinction horizon (Figure 2, purple trace). In older bins it usually overlapped with one or both former treatments, but retention was consistently lower than either.

The Strong treatments (Figure 2, blue and orange traces respectively) showed great loss of branching history in all bins compared to the aforementioned treatments. In all multiple comparison tests, the Strong treatments almost always formed a group to the exclusion of the other treatments, except at 35,000–40,000 updates (as data was lacking here across all treatments). Most notable are the large differences from the other treatments in bins older than 35,000–40,000 updates. Compared to Strong Pulse, Strong Press showed generally lower persistence of nodes in the oldest bins, and generally higher persistence in bins from 65,000–70,000 updates onwards. However, the Strong treatments did not differ significantly in any bin, and showed much wider variation than the other treatments. This was due to many fewer nodes in the oldest bins (Figure S5).

### Effects on Tree Stemminess–Pybus-Harvey Gamma

**Figure 3:**
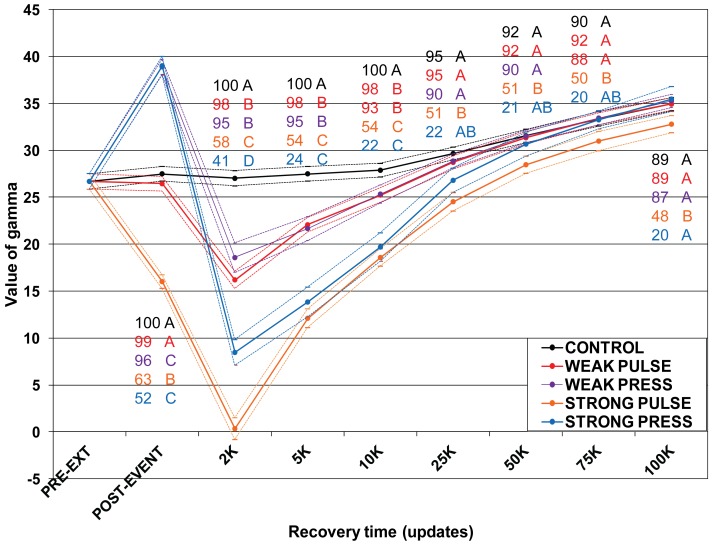
Pybus-Harvey gamma (PHG) at select time points after the extinction episode. The y-axis is PHG. Data are averages plus/minus two standard errors. Differences between treatments were tested with one-way ANOVA followed by Tukey-HSD corrected multiple comparison tests. Letters indicate treatments that are NOT significantly different from each other after correction for multiple comparisons, and are colour-coded by treatment. Numbers with each letter indicate number of replicates used for test. Only replicates that maintained the pre-extinction root are included at each time point.

Using PHG, the four treatments responded differently to the extinction, but behaved similarly afterwards (Figure 3). In controls, PHG values increased monotonically over the course of the experiment, similar to previous observations in branching model studies [23]. PHG values initially declined for both Pulse treatments - although this was due to reduced population size – and continued over the first 2000 updates of recovery before reversing. On average, the decline was more extreme for Strong Pulse, with PHG sometimes going negative. In both press treatments, PHG values increased sharply during the press episode, rising to a maximum around the midpoint before leveling off or even falling slightly. PHG values then declined during the early phase of the recovery, but remained positive (in replicates that lost the pre-extinction root, PHG sometimes went negative). This unexpected result requires explanation. In Data S4, we show that this effect results from an increase in genotypic turnover that occurs during the press episode, but relaxes back to its previous regime when pre-extinction conditions are restored (Figure S6).

Over the course of the recovery, all four extinction treatments converged to average PHG values near those for Control. Only Strong Pulse, which showed the steepest average decline in PHG, remained statistically distinct over the length of the recovery period. Extended experiments with Control and Strong treatments showed that all treatments would eventually converge to roughly the same average PHG value (not shown).

### Effects on Tree Stemminess–Noncumulative Astemminess

**Figure 4:**
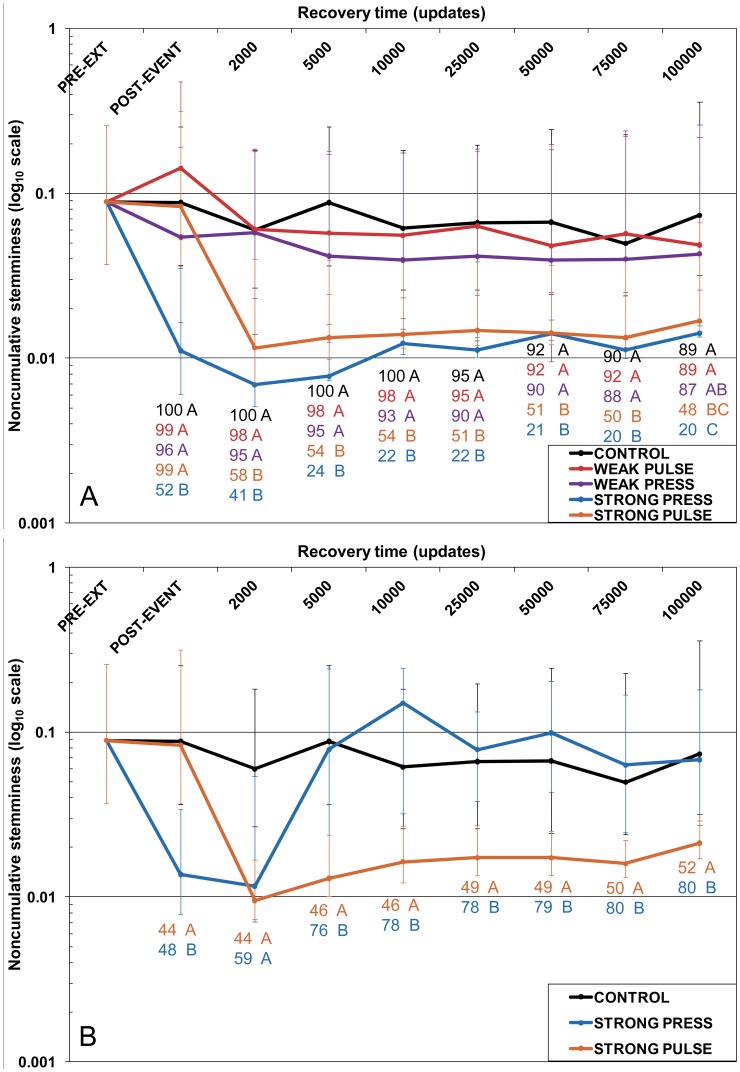
Noncumulative stemminess (NCS) at select time points after the extinction episode. The y-axis is NCS (plotted on a log_10_ scale). Data are median NCS values with 25% and 75% interquartiles. a) Replicates where the pre-extinction phylogenetic root was retained over the duration of the recovery period. b) Strong press (blue trace) and strong pulse (orange trace) replicates where the pre-extinction phylogenetic root was lost during either the extinction itself or the recovery period. The black trace is the same as in panel a) (for comparison purposes). Differences between treatments were tested with Kruskal-Wallis tests followed by Tukey-HSD corrected multiple comparison tests. Letters indicate treatments that are NOT significantly different from each other after correction for multiple comparisons, and are colour-coded by treatment. Numbers with each letter indicate the number of replicates used for testing.

Using NCS, the Strong extinctions left clear long-term signatures in tree stemminess compared not only to the immediate pre-extinction state, but also to Control and Weak treatments (Figure 4a, b). However, the Strong treatments showed different effects depending on retention of the pre-extinction root.

**Figure 5:**
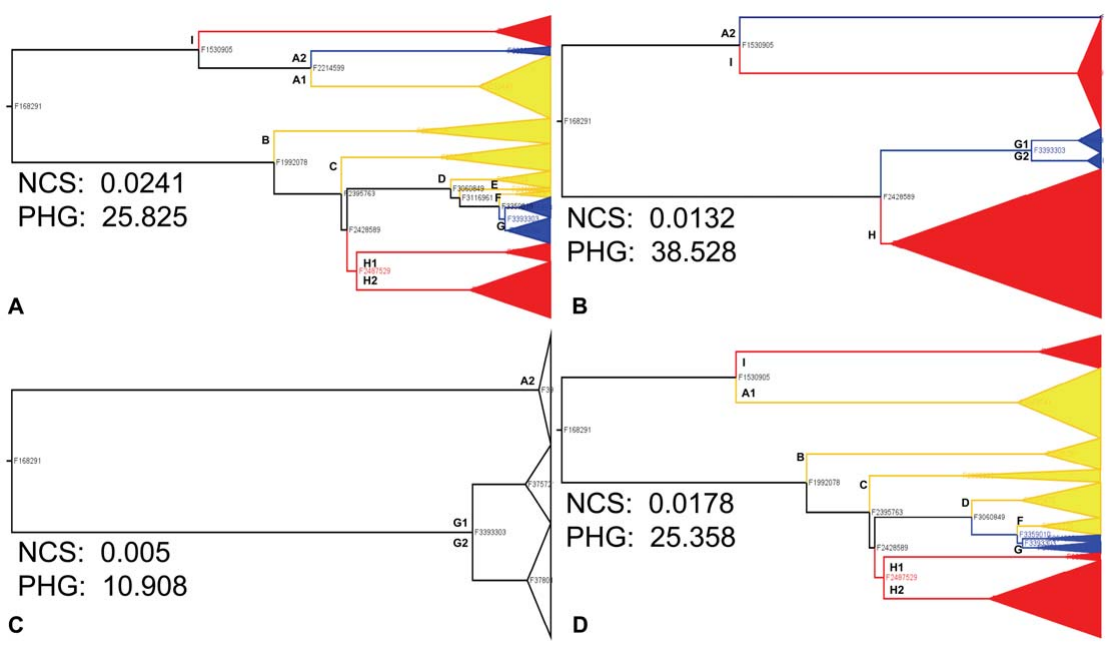
Example phylogenetic trees showing loss of pre-extinction history during and after press episode, from a replicate population that maintained the pre-extinction phylogenetic root. Node labels are Avida genotype IDs. Branch lengths are scaled in Avida absolute time. a) Immediate pre-extinction tree. Clades highlighted in yellow are lost during press episode; clades highlighted in red survive press episode but are lost during recovery. Clades highlighted in blue are those that comprise the tree in panel c). Organisms in clades D, E, F, G contain same phenotypic profiles as those in clade C (not shown due to space constraints). b) Tree from end of press episode. Clades highlighted in red are lost during recovery. c) Tree from 2000 updates into recovery after press episode (107,000 updates). d) Control tree from 107,000 updates, showing retention of clades that were otherwise lost in the press extinction and recovery.

In Strong Press replicates that retained the pre-extinction root, median NCS values declined during both the press episode and early recovery, indicating a shift towards trees with longer distances from the root to the next-oldest internal nodes (Figure 4a, blue trace). The recovery could further amplify this behaviour (see the Strong Press example in Figure S2). NCS continued to decrease even into the early recovery, indicating additional loss of interior nodes nearer the root. Figure 5 shows a clear example: following the loss of several deeper-branching clades during the press episode itself (Figure 5a, yellow-highlighted clades), additional loss occurs during the early recovery (Figure 5a, b, red-highlighted clades), leaving a tree where the next oldest branching events lie much farther from the root, and are the divergence points for what would otherwise have remained small clades (Figure 5d, blue-highlighted clades).

Strong Pulse increased the median NCS value slightly, but NCS declined quickly during the early recovery (Figure 4a, b orange traces). By 2000 updates of recovery, median NCS values for Strong Pulse were similar to those for Strong Press. However, the interquartile ranges for Strong Pulse were wider and more asymmetric. These trends persisted in both Strong treatments over the remainder of the recovery. Weak Pulse had no obvious effect on median NCS values, indicating the basic stemminess properties of phylogenies remained largely unaltered (Figure 4a, red trace). Median NCS values for Weak Press did not differ significantly from the former two treatments, but were consistently lower, suggesting that deep history was affected to a greater extent (Figure 4a, purple trace).

When the pre-extinction root was lost during either the extinction episode or the recovery, the Strong treatments in particular showed divergent behaviour (Figure 4b). The same general trend towards persistently lower NCS values was observed for Strong Pulse, though much less variable. For Strong Press, median NCS values initially declined during the press episode and early recovery, then generally increased to control levels where the pre-extinction root was retained (Figure 4a, b, black trace), though highly variable (Figure 4b, blue trace and error bars).

## Discussion

In this study, we used model communities of digital organisms to examine effects of two principal types of mass extinction on phylogenetic structure and demonstrated that:

1. Strong Press extinctions resulted in a significant shift towards phylogenetic roots that originated during or after the extinction episode (Figure 1). This effect was not observed for other treatments.
2. Populations subjected to Strong treatments experienced greater loss of phylogenetic branching history as compared to Control and Weak treatments. Loss of branching history was accelerated during the Strong Press episode, with deep structure of the trees being eroded to a greater degree vs. the same time period in Control. Significant differences between Control and Weak treatments were best seen where branching events were highly concentrated in the phylogenies. Differences between Strong treatments were suggested, but not significant.
3. Extinction produced complex changes in tree stemminess. While the Pybus-Harvey gamma statistic (PHG) only showed clear differences between treatments in the early stages of recovery, by noncumulative stemminess (NCS) clear long-term phylogenetic signatures were left relative to both the pre-extinction state and Control experiments.

### Strong Random and Strong Selective Extinction Leave different Distributions of Root Ages

The most basic way in which we measured loss of history here is a shift in the age of the pre-extinction population’s phylogenetic root. This means that one or more of the most basal clades in the tree has gone extinct, with a younger branching event replacing it. When measured this way, the two strong extinction regimes left distinct effects on deep history. Strong Press resulted in the greatest amount of root replacement, both as a result of the extinction episode itself and the early recovery (Figure 1). Strong Pulse did not produce this result even by the end of the experiment. By this measure, Strong Press was the most damaging of the treatments, although we emphasize that the final result was shaped by both the extinction and the recovery.

Phylogenetically nonrandom extinction has been shown to erode more of a clade’s evolutionary history than purely random extinction [4], [5], [7], [15], [17], [51]. While this can be explained prosaically (phylogenetically clustered extinctions “delete” more of the inner history of a tree), other forces are at play in our system. During a press episode, conditions shift to favouring organisms that maximize base replication at the expense of ecological functionality, which take over the population and drive less well-adapted organisms (and eventually the entirety of clades they are members of) extinct. Survivors enter the recovery on a new ecological and genetic “playing field” [21]. Further, genotype-level turnover increases (shown in Data S4), and deep history is lost even before the onset of recovery. Additional loss of deeper-branching clades during the recovery occurs often as communities re-adapt to the restored environment, potentially erasing remaining pre-extinction history (except for the lineage of the post-extinction root), (Figure 1). Extinction of these “dead clades walking” [52] may result from either drift or selectivity during the recovery [29].

Strong Pulse did not produce the same radical shift in the distribution of root ages, even though the mode of deep roots was reduced considerably by the initial event and basal clades continued to be lost over the course of the recovery. This may be because the pulse event itself thins out “higher taxa”, and during the recovery period background extinction further removes deeper-branching clades. Survivors picked at random also do not experience the genomic and phenotypic changes incurred by press survivors [21], and expand into a much emptier population space where they experience decreased competition for supplied resources. Critically, this sorting of the winners and losers of the extinction continues during the recovery period, and can be as important as the extinction events themselves in determining the phylogenetic characteristics of surviving clades [53], [54].

### Temporal Binning of Nodes Fails to Detect Significant differences between Strong Extinctions

Root age shift is a coarse measure that is uninformative about more subtle variation in tree structure. In these digital communities, most of the branching events are concentrated near the leaves of the tree (Figure S5, see also Methods), so when a phylogeny is temporally binned, bins nearest the tips contain the most information, and differences in loss of history will be least ambiguous. In these, Control (representing background extinction) and Weak treatments showed significant differences from each other in percent retention of nodes. Weak Press showed the lowest percent retention, consistent with previous findings that selective extinction exacerbates loss of history when compared to random extinction of similar magnitude [4], [15], [17], [55]. However, we caution that the calibration of Weak Press to Weak Pulse is highly imperfect. In the oldest bins, which contain many fewer nodes, these three treatments did not differ significantly. However, Weak Press again has consistently lower retention, and we interpret this to mean that nodes in these bins are slightly more likely to be lost with this treatment than under background or weak random extinction. While this result suggests that the oldest nodes in a phylogeny will generally be resistant to weak extinction, our observations are likely dependent on the particular tree structure of these model communities.

Although this analysis demonstrated genuine phylogenetic consequences of the press episodes, it detected no significant differences between the two Strong treatments (Figure 2), suggesting that they were equally destructive to deep tree structure (except when all pre-extinction history was lost). Inspection of the data suggests that Strong Press showed lower retention in the deepest bins, with slightly higher retention than Strong Pulse in some later bins. This is consistent with the press-induced shift in root ages, which resulted in some trees with quite late-originating roots (Figure 1b, post-event distribution). Strong Pulse almost always produced four-leaf trees that were constrained to contain only one or two nodes in any bin. These trees showed higher retention in the oldest bins, and the converse in bins nearer the extinction horizon. This is consistent with the lesser reduction of deep roots, and fewer post-extinction trees with very late-originating roots, compared to Strong Press (Figure 1c, post-event distribution). However, a conservative interpretation of the statistics suggests no clear differences between the Strong treatments. From this perspective, extinction magnitude was more important than type in determining the extent to which deep history was affected. Our interpretation is that in terms of effects on phylogenetic history, a press extinction severe enough to cause ecosystem collapse would be equivalent to a random extreme bottleneck.

One possible anomaly in these results is the unexpected stemminess of the trees, with long basal branches and most branching events near the tips. In retrospect, this may be plausibly explained by the decoupling of cladogenesis and anagenesis in Avida, consistent with behaviour observed in BPMs [44], and certainly not unique to digital organisms. However, this effect on the tree topology may obfuscate the differences between treatments.

### Failure of PHG to Detect Long-term Signatures of Mass Extinction

PHG has become the “industry standard” for rejecting constant rates of diversification in molecular phylogenies. As originally described by Pybus and Harvey [47], more negative values of PHG indicate nodes are closer to the root, while more positive values indicate nodes are closer to the tips of the tree. It is thus tempting to view PHG as a kind of stemminess metric, able to help distinguish among trees that result from different evolutionary processes. However, our results demonstrate that PHG cannot detect long-term signatures of mass extinction that are evident through other means (e.g. NCS). PHG shows clear differences between treatments during both the extinction event itself and the early phases of the recovery, but these differences diminish as the recovery progresses. This may simply be because PHG is strongly influenced by divergences near the leaves of the tree, while NCS is strongly influenced by divergences near the root, so the latter metric will be more informative for trees that have very long distances between the root and the next oldest internal nodes.

Alternatively, Liow et al. [23] point out that PHG actually detects whether or not there is any change in the rate of diversification over time. A significantly negative PHG results from a decreasing rate of diversification, while a positive or non-significantly negative value may be due to i) slowly decreasing diversification, ii) exponential diversification, or iii) turnover at constant diversity. It seems likely the last is the case by the end of the experiments (and in the extended runs), with the communities having already reached a dynamic equilibrium where no real net change in diversification rate occurs. Thus, PHG can detect differences between treatment types only before (or shortly after) dynamic equilibrium has been reached, which is during the earliest stages of the recovery.

For most real cases, we do not know where in its evolutionary trajectory a clade is, nor can we know outcomes under alternative histories. Thus, we cannot solely use metrics such as NCS or PHG to infer past history of mass extinction for a given molecular phylogeny. Other processes, such as emergence of a new group that selectively sweeps a population even without external perturbation, could produce patterns similar to the ones we observed. Additional information from the geological record would then be necessary to implicate mass extinction [12]. In our experiments, we can recognize a long-term signature of mass extinction with NCS because both the immediate pre-extinction and untreated control states are available for comparison, and we know the timing and nature of the extinction events. PHG fails to show long-term differences even with this extra information available, demonstrating unsuitability of this metric for situations involving major changes in branch lengths and tree structure over time. These results argue against the general utility of the PHG/molecular phylogeny combination for examining consequences of, or drawing conclusions about, evolutionary processes that have operated very far in a clade’s past.

### Differences between Random and Selective Mass Extinction with NCS Depend on Root Age

Unlike PHG, NCS detected clear differences between Strong and Weak extinctions, but not between Weak extinctions and control. A substantial amount of phylogenetic structure can survive even a 90–95% random loss of tips [7], so this result is not unexpected. In the present results, mass extinction only makes a clear difference when so severe that the deep structure of the tree is heavily compromised, as shown by the shift towards trees with longer basal branches when the pre-extinction root persists (Figure 4a). Additional changes during the early recovery (Figs. 4 and 5, Figs. S2 and S3) also reinforce the conclusion above (for root age) that the recovery can be as influential as the extinction in shaping phylogenetic characteristics.

The Strong extinctions were of comparable magnitude in their effects on the temporal structure of the phylogeny (Figure 2, Figure S5). The NCS results seem to reinforce our conclusion above that extinction magnitude matters more than type, but some re-consideration is warranted. When the pre-extinction root was retained, no difference in median tree stemminess was detectable, but the variability in NCS was much higher for Strong Pulse than for Strong Press. This increased variance in Strong Pulse NCS scores results from a greater tendency to retain deeper nodes compared to Strong Press, giving rise to some trees with higher NCS scores (short branches near the root inflate the value of NCS). By contrast, Strong Press trees that retain the pre-extinction root tend to have more uniformly long basal branches. We verified this disparity by examining the distance between the root and the next two oldest nodes in the end-experiment trees (Table 1). The median distance between the root and the next oldest node does not differ significantly between Strong Press and Strong Pulse, but the latter has much larger interquartiles. Weak Press results do not differ significantly from either Weak Pulse or Control, but the median distances of the next two oldest nodes from the root are somewhat larger, again indicating slightly greater tendency towards loss of deep-branching clades. Thus, when the pre-extinction root is retained, the difference between Strong Pulse and Strong Press extinction is mostly a difference in variability not central tendency.

**Table 1:** Median age of root of the end-experiment phylogenetic trees, and the median distances (in updates) from the root to the first and second next oldest nodes in the tree, for replicate populations in which the pre-extinction root was retained.

Treatment	Median age of root (updates)	25%	75%	Median root-1st node distance (updates)	25%	75%	Multiple comparison grouping	Median root-2nd node distance (updates)	25%	75%	Multiple comparison grouping
CONTROL (89)	854	586	1496	4349	616	14098	A	23459	8419	72604	A
WEAK PULSE (89)	854	586	1419	4349	705	12491	A	27464	8419	77344	A
WEAK PRESS (87)	854	591.5	1416.5	6694	612.5	23010.5	A	35076	8535.5	101256.5	A
STRONG PULSE (48)	907	618.25	1443.25	96161.5	7867.597	98894.75	B	99952	98850.5	114495.5	B
STRONG PRESS (20)	830	574	1297.25	103537.5	84233.75	104152.5	B	104489.5	104246.3	113613.8	B

Differences between treatments were tested with a Kruskal-Wallis test, followed by a Tukey-corrected multiple comparison test. Letters in “Multiple comparison grouping” indicates treatments that are NOT significantly different after correction for multiple comparisons.

When the pre-extinction root is lost, Strong Press replicates have larger NCS values and wider interquartiles (Figure 4b, Table S2). Particularly during the early recovery, NCS increasingly measures trees with roots and deep nodes that originate during (or after) the press episode (Table S1), due to additional loss of clades branching from the previous root. The strong shift towards much later-originating roots does not occur in Strong Pulse (Table 1, Table S2), and the reduced variation in NCS indicates more uniformity of deep tree structure (as with Strong Press replicates that retained the pre-extinction root). Taken together, these results suggest that strong random extinction (the pulse) will, on average, have qualitatively similar effects on tree stemminess regardless of root loss (orange traces in Figure 4a,b), whereas strong selective extinction (the press) shows different effects depending on retention of the root (blue traces in Figure 4a,b). We again emphasize these differences result from a combination of events during the extinction itself and the recovery period.

### Concluding Remarks

Our detection of long term signatures left in phylogenetic trees in turn suggests that mass extinctions which were more ecologically damaging [56] may have been more destructive to the deep phylogenetic history of clades that survived the extinction and subsequently rediversified. There is some paleontological evidence for this – the terebratulide brachiopods on either side of the Frasnian-Fammenian extinction demonstrate that the most basal members of a clade can suffer disproportionately, leaving only more derived taxa as post-extinction survivors [20] – but further testing will require a battery of well-resolved phylogenies whose roots are known to lie earlier than a particular extinction event. The present results may also have bearing on how the modern biota may be phylogenetically reshaped if current anthropogenic extinctions come to rival past mass extinctions in scope: whether on the one hand surviving taxa with deep shared ancestry but very “hollowed-out” phylogenies are retained, or whether taxa that may be ecologically diverse but have diverged relatively recently, having lost most (if not all) of the basal members of their clades.

## Supporting Information

Figure S1 Schematic of the cascading trophic interactions used in this study (goes with Data S1). Resources are associated with each of the logic functions shown. The reward value for performing the particular function is shown in parentheses next to the function name. A line connecting resources signifies that an organism performing the lower-level function consumes the incoming resource and produces a by-product that is available for any organism that can perform the higher-level function. Only the resources associated with the lowest-level functions NOT and NAND are provided exogenously. Example conversion factors are shown to the right of one of the inflowing resources and on the connection arrows; in this case, three units of the NOT resource are required to produce one unit each of the resources for AND, ORN, and OR. Similarly, three units of the AND resource are required to produce one unit each of the ANDN, NOR, and XOR resources.
(TIF)

Figure S2 Change in PHG vs time for a representative i) control experiment (black trace), ii) strong pulse experiment (orange trace), iii) strong press experiment (blue trace) where the pre-extinction MRCA was retained. Time corresponding to press episode is highlighted by blue box. The control curve comes from a different replicate population, and is included to illustrate the behaviour of PHG resulting from no perturbation.
(TIF)

Figure S3 Change in NCS vs. time for a representative i) control experiment (black trace), ii) a strong pulse experiment (orange trace), iii) a strong press experiment (blue trace), where the pre-extinction MRCA was retained. Time corresponding to press episode is highlighted by blue box. All three series have the same pre-extinction history.
(TIF)

Figure S4 Graphical representation of loss of branching history, using lineage-through-time (LTT) data visualized as histograms (goes with Data S2). The x-axis is bins of node ages up to 100,000 updates, the y-axis is number of nodes in the bin. Each bin represents a width of 10,000 updates. Nodes are dated using age in updates. A node whose age falls between time *t* and (*t*+10,000) is placed in the appropriate bin. Since the last bin (95,000–100,000 updates) always contains many more nodes than older bins, the y-axis has been truncated to permit visualization of bars in older bins. Panels a-d) LTT histograms for a representative control experiment at a) immediate pre-extinction, b) 101500 updates, c) 102500 updates, d) 105000 updates. Reduction of bar height in most recent bin cannot be seen due to y-axis truncation. Panels e-h) LTT histograms for the same absolute time points in the corresponding replicate population during a strong press episode. Panel e) is identical to panel a) above.
(TIF)

Figure S5 Plot of number of nodes per bin for temporally binned LTT data (supports main text Figure 2). Average number of nodes present per bin. Y-axis now shows number of nodes per bin, rather than percent retention, and is plotted on a log_2_ scale. Pre-extinction data (underlined text in multiple comparison groupings) are included for direct comparison with treatment data. Statistical treatment of data is as described for main text Figure 2.
(TIF)

Figure S6 Differing origination/extinction dynamics of genotypes underlie behaviour of PHG in press vs. pulse extinctions (goes with Data S4). In panels a and c, blue series–originations, red series–extinctions, black series–persistences. a) Origination/extinction dynamics for a representative strong press experiment. b) Corresponding change in PHG vs. time for panel a). c) Origination/extinction dynamics for a representative strong pulse experiment. d) Corresponding change in PHG vs. time for panel c).
(TIF)

Table S1 Contains Supplementary Table S1.
(DOC)

Table S2 Contains Supplementary Table S2.
(DOC)

Data S1 General introduction to Avida and cross-feeding relationships.
(DOC)

Data S2 Using lineage-through-time data to assess erosion of evolutionary history in phylogenies.
(DOC)

Data S3 Definitions of stemminess metrics Pybus-Harvey gamma (PHG) and noncumulative stemminess (NCS).
(DOC)

Data S4 Changes in Pybus-Harvey gamma linked to changes in genotypic turnover.
(DOC)

File S1 Source code for Avida v. 2.1 and configuration files for performing the experiments described in the main text.
(RAR)

File S2 Source code for the TreeLoader software used to convert Avida population data into phylogenetic trees and calculate tree shape metrics PHG and NCS.
(RAR)
