# Supplementary figures and images for: A Comparison of the Effects of Random and Selective Mass Extinctions on Erosion of Evolutionary History in Communities of Digital Organisms
Source: PLoS One. 2012 May 31;7(5):e37233. doi: 10.1371/journal.pone.0037233 (PMC3365035; doi:10.1371/journal.pone.0037233)

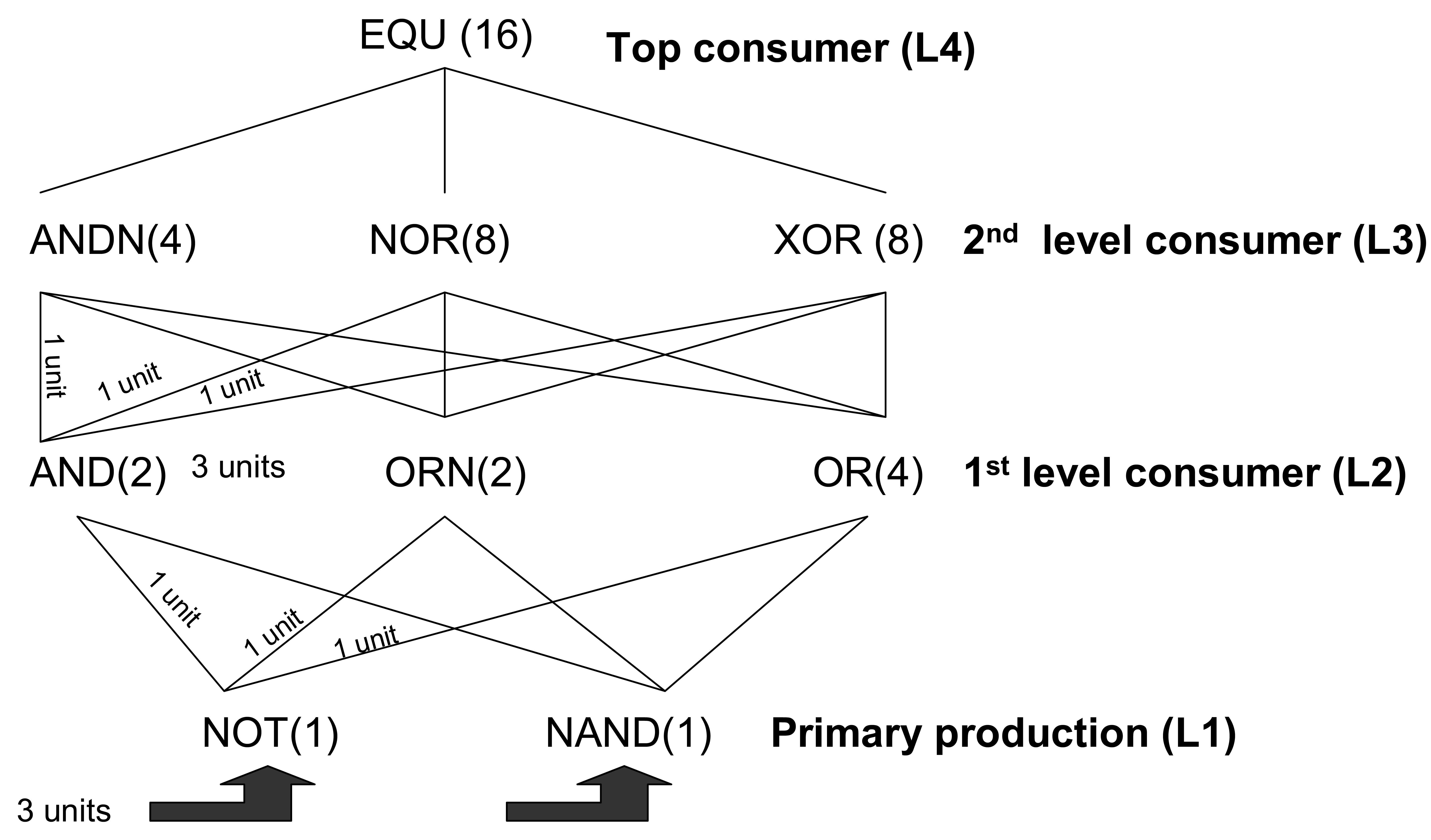

Supplement: Figure S1 — Schematic of the cascading trophic interactions used in this study (goes with Data S1). Resources are associated with each of the logic functions shown. The reward value for performing the particular function is shown in parentheses next to the function name. A line connecting resources signifies that an organism performing the lower-level function consumes the incoming resource and produces a by-product that is available for any organism that can perform the higher-level function. Only the resources associated with the lowest-level functions NOT and NAND are provided exogenously. Example conversion factors are shown to the right of one of the inflowing resources and on the connection arrows; in this case, three units of the NOT resource are required to produce one unit each of the resources for AND, ORN, and OR. Similarly, three units of the AND resource are required to produce one unit each of the ANDN, NOR, and XOR resources. (TIF) [file pone.0037233.s001.tif]

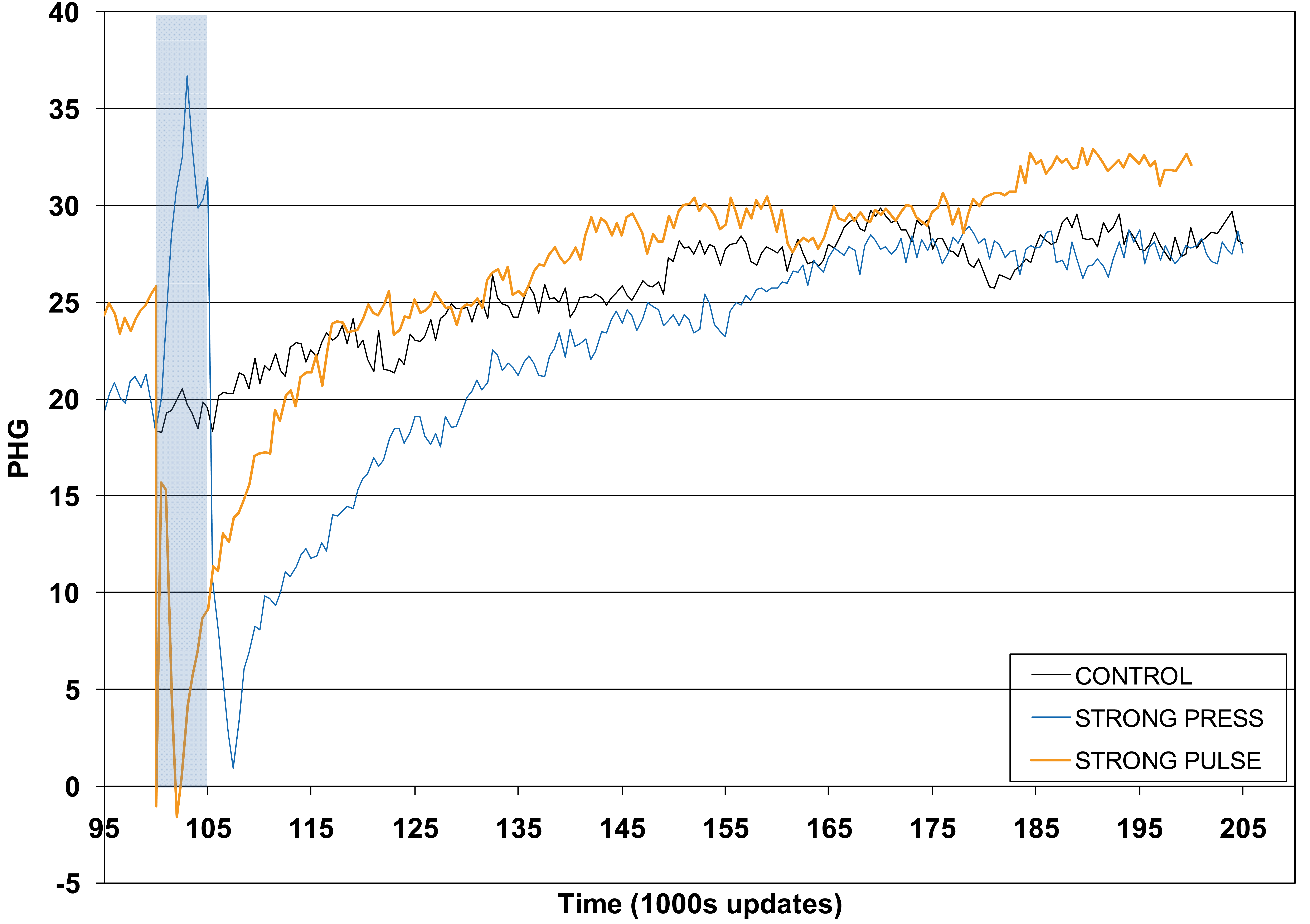

Supplement: Figure S2 — Change in PHG vs time for a representative i) control experiment (black trace), ii) strong pulse experiment (orange trace), iii) strong press experiment (blue trace) where the pre-extinction MRCA was retained. Time corresponding to press episode is highlighted by blue box. The control curve comes from a different replicate population, and is included to illustrate the behaviour of PHG resulting from no perturbation. (TIF) [file pone.0037233.s002.tif]

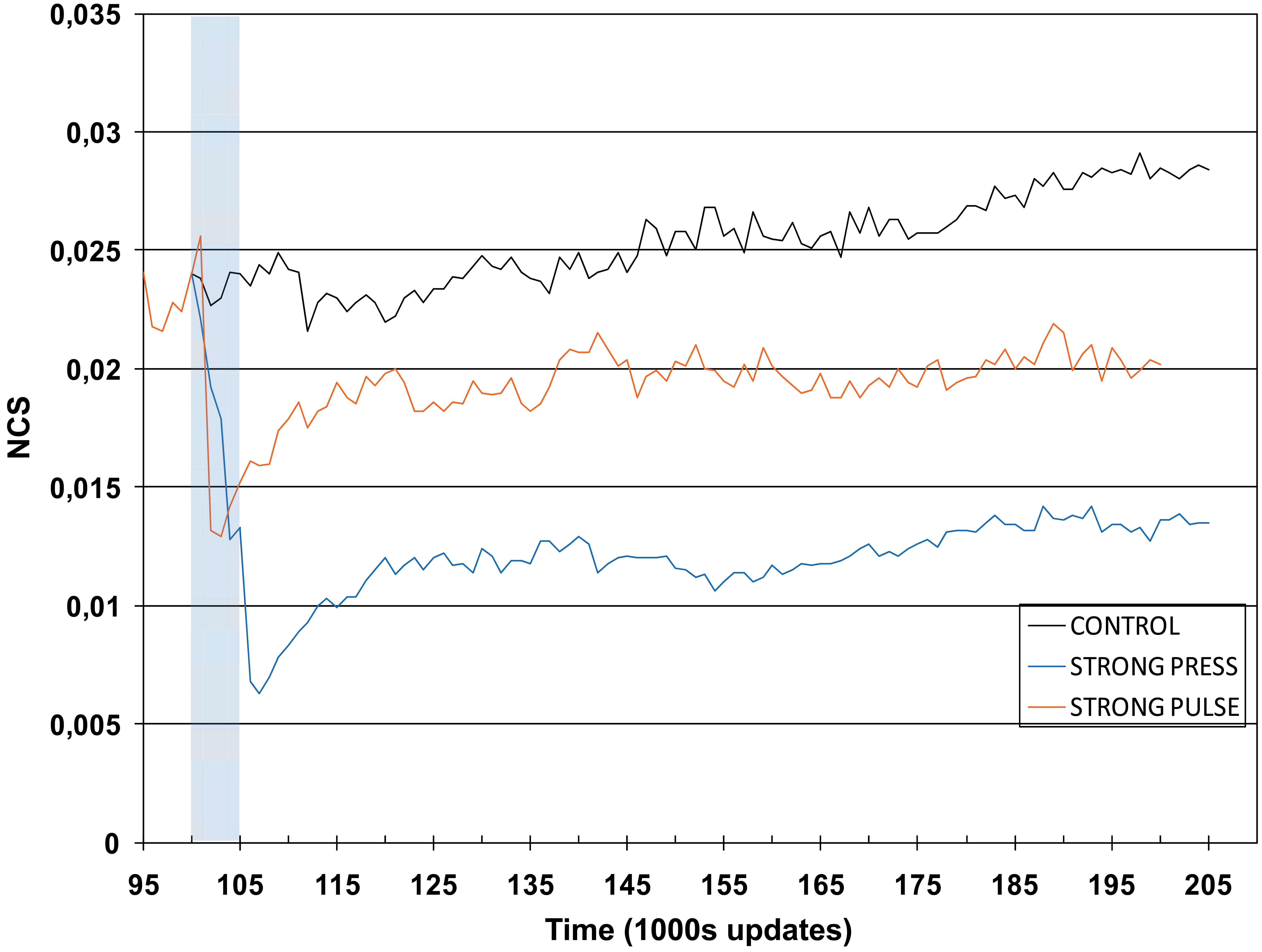

Supplement: Figure S3 — Change in NCS vs. time for a representative i) control experiment (black trace), ii) a strong pulse experiment (orange trace), iii) a strong press experiment (blue trace), where the pre-extinction MRCA was retained. Time corresponding to press episode is highlighted by blue box. All three series have the same pre-extinction history. (TIF) [file pone.0037233.s003.tif]

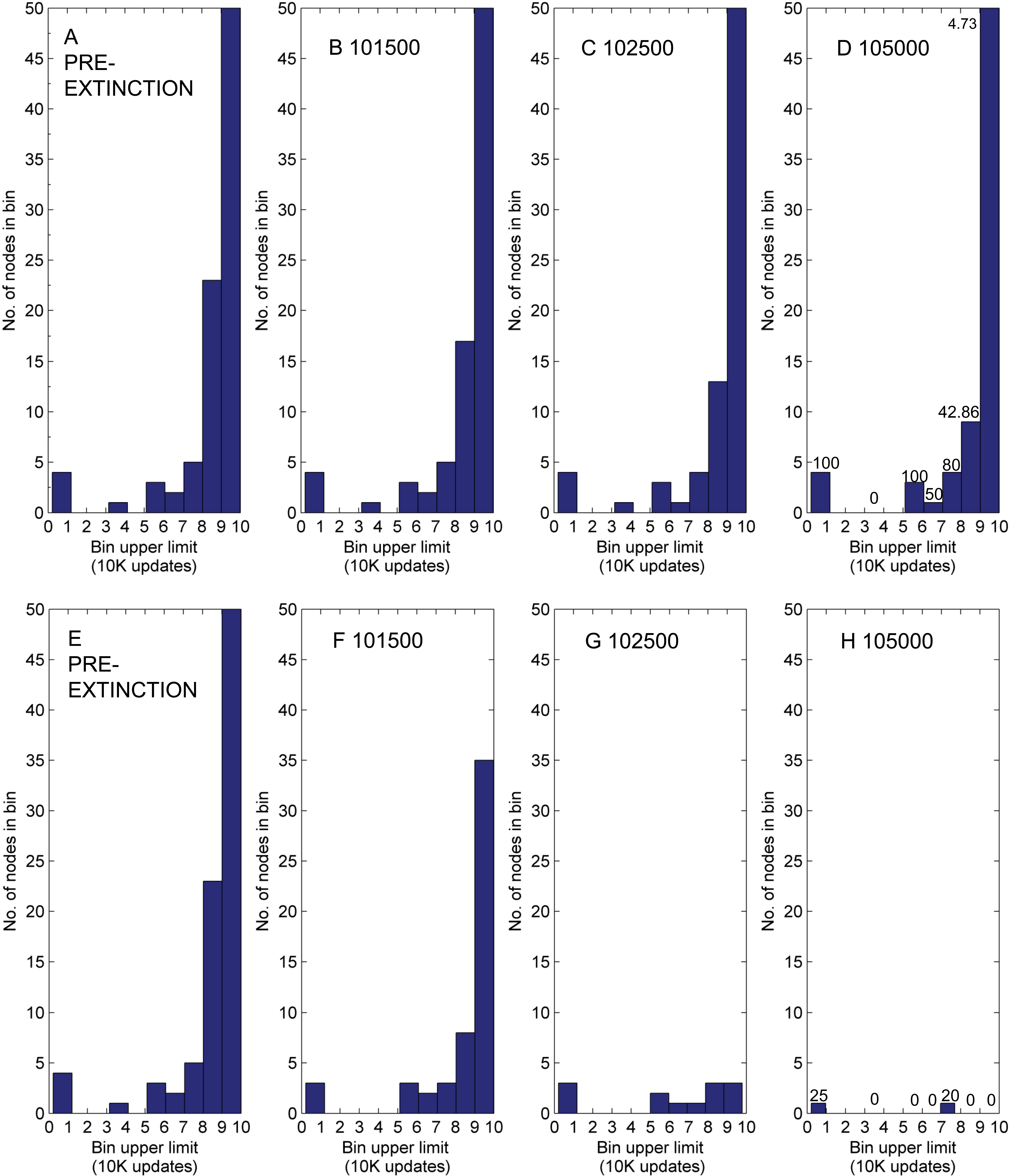

Supplement: Figure S4 — Graphical representation of loss of branching history, using lineage-through-time (LTT) data visualized as histograms (goes with Data S2). The x-axis is bins of node ages up to 100,000 updates, the y-axis is number of nodes in the bin. Each bin represents a width of 10,000 updates. Nodes are dated using age in updates. A node whose age falls between time t and (t+10,000) is placed in the appropriate bin. Since the last bin (95,000–100,000 updates) always contains many more nodes than older bins, the y-axis has been truncated to permit visualization of bars in older bins. Panels a-d) LTT histograms for a representative control experiment at a) immediate pre-extinction, b) 101500 updates, c) 102500 updates, d) 105000 updates. Reduction of bar height in most recent bin cannot be seen due to y-axis truncation. Panels e-h) LTT histograms for the same absolute time points in the corresponding replicate population during a strong press episode. Panel e) is identical to panel a) above. (TIF) [file pone.0037233.s004.tif]

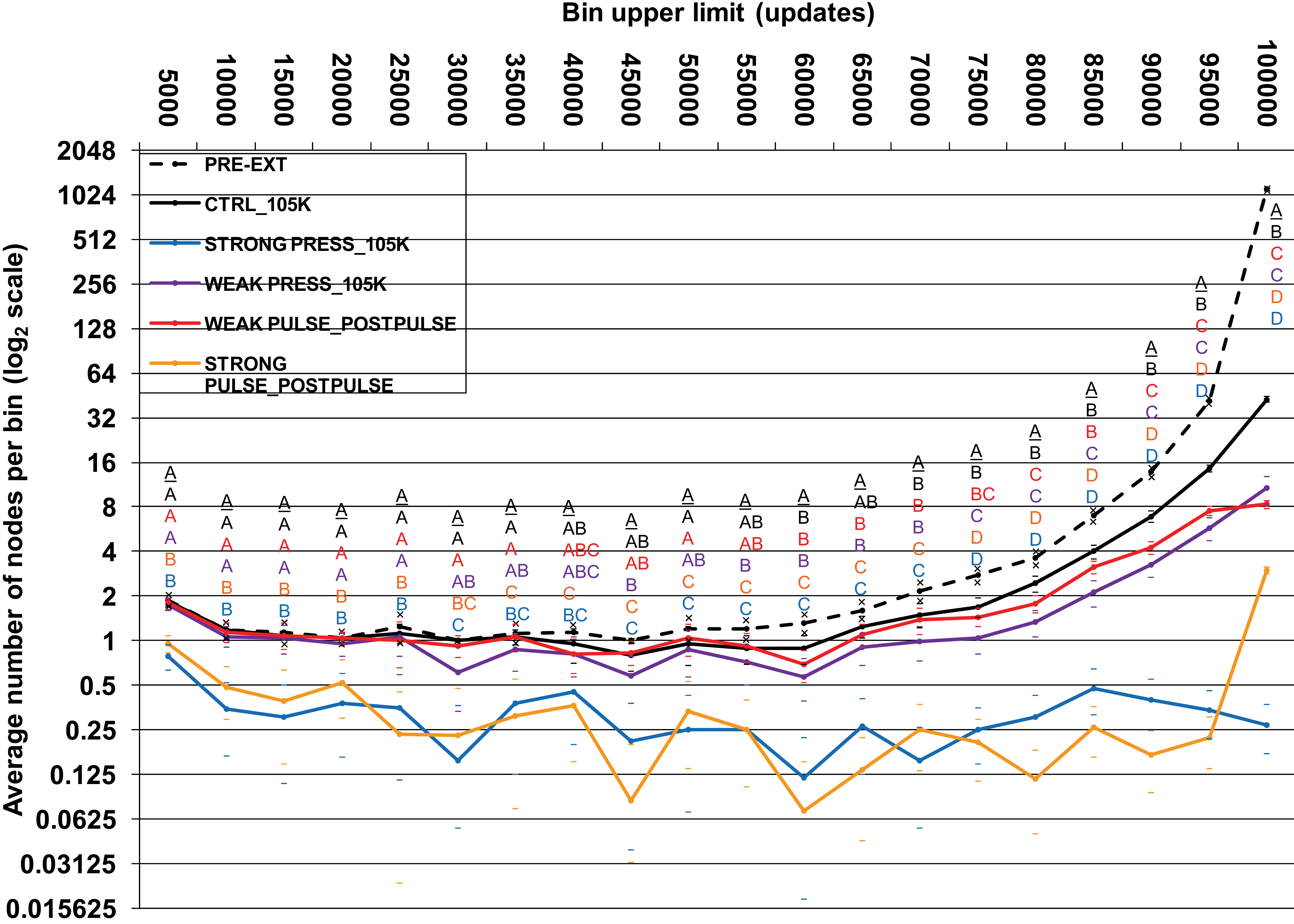

Supplement: Figure S5 — Plot of number of nodes per bin for temporally binned LTT data (supports main text Figure 2). Average number of nodes present per bin. Y-axis now shows number of nodes per bin, rather than percent retention, and is plotted on a log2 scale. Pre-extinction data (underlined text in multiple comparison groupings) are included for direct comparison with treatment data. Statistical treatment of data is as described for main text Figure 2. (TIF) [file pone.0037233.s005.tif]

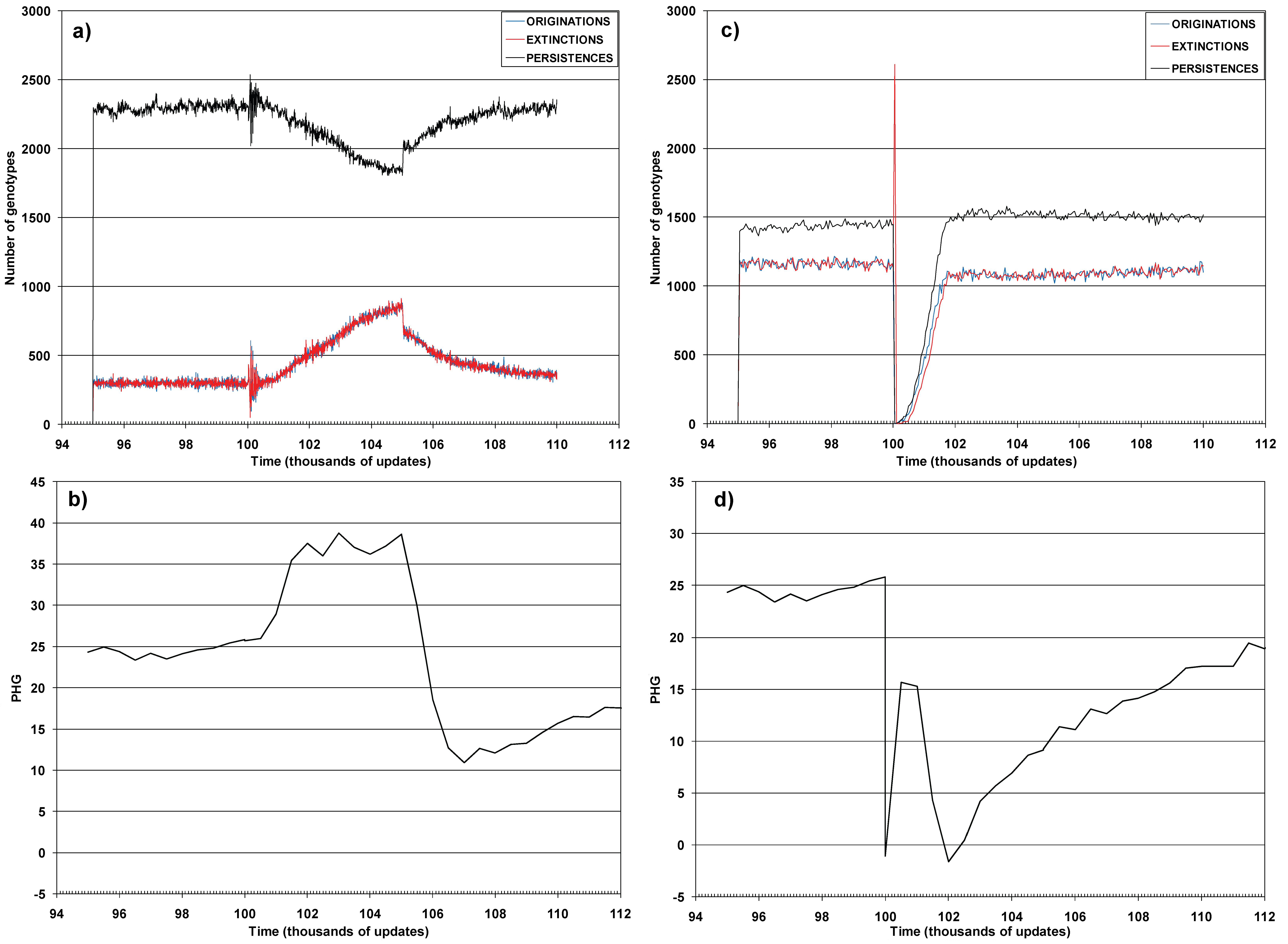

Supplement: Figure S6 — Differing origination/extinction dynamics of genotypes underlie behaviour of PHG in press vs. pulse extinctions (goes with Data S4). In panels a and c, blue series–originations, red series–extinctions, black series–persistences. a) Origination/extinction dynamics for a representative strong press experiment. b) Corresponding change in PHG vs. time for panel a). c) Origination/extinction dynamics for a representative strong pulse experiment. d) Corresponding change in PHG vs. time for panel c). (TIF) [file pone.0037233.s006.tif]
