# Supplementary material for: A Comparison of the Effects of Random and Selective Mass Extinctions on Erosion of Evolutionary History in Communities of Digital Organisms
Source: PLoS One. 2012 May 31;7(5):e37233. doi: 10.1371/journal.pone.0037233 (PMC3365035; doi:10.1371/journal.pone.0037233)
Supplement: Table S1 — Contains Supplementary Table S1. (DOC) [file pone.0037233.s007.doc]

**Table S1. Loss of branching nodes from phylogenetic trees in response to press episode. Data are averages from all 100**

**replicate populations. Only nodes originating before the extinction event are included for time points after pre-extinction.**

**Phylogenetic roots were not included in the totals.**

|  | Pre-extinction | Control, press episode midpoint | Control, end press episode | Weak press, press episode midpoint | Weak press, end press episode | Strong press, press episode midpoint | Strong press, end press episode | Weak pulse, post-event | Strong pulse, post-event |
| --- | --- | --- | --- | --- | --- | --- | --- | --- | --- |
| Average | 1190.11 | 154.76 | 77.53 | 112.30 | 28.81 | 22.99 | 3.68 | 33.82 | 1.99 |
| Std. Error | 12.315 | 2.565 | 1.44 | 4.26 | 2.20 | 1.7 | 0.295 | 0.115 | 0.01 |
| L95% | 1165.37 | 149.63 | 74.65 | 103.78 | 24.41 | 19.59 | 3.09 | 33.59 | 1.97 |
| U95% | 1214.63 | 159.89 | 80.41 | 120.82 | 33.21 | 26.39 | 4.27 | 34.05 | 2.01 |
