# Supplementary material for: A Comparison of the Effects of Random and Selective Mass Extinctions on Erosion of Evolutionary History in Communities of Digital Organisms
Source: PLoS One. 2012 May 31;7(5):e37233. doi: 10.1371/journal.pone.0037233 (PMC3365035; doi:10.1371/journal.pone.0037233)
Supplement: Table S2 — Contains Supplementary Table S2. (DOC) [file pone.0037233.s008.doc]

Table S2. Median age of root of the end-experiment phylogenetic trees, and the median distances (in updates) from the root to the first and second next oldest nodes in the tree, for replicate populations in which the pre-extinction root was lost.

| Treatment  (no. of replicates) | Median age of root (updates) | 2.5% | 97.5% | Median distance root-1st node  (updates) | 2.5 % | 97.5 % | Median distance root-2nd node  (updates) | 2.5 % | 97.5 % |
| --- | --- | --- | --- | --- | --- | --- | --- | --- | --- |
| STRONG PULSE (52) | 36738 | 760.475 | 99922.33 | 49650 | 205.725 | 112729.9 | 71906 | 2674.7 | 123664.9 |
| STRONG PRESS (80) | 104823.5 | 940.4 | 109654.2 | 3040.5 | 19.925 | 104166.6 | 24303 | 376.075 | 120046.1 |
