# Supplementary material for: A Comparison of the Effects of Random and Selective Mass Extinctions on Erosion of Evolutionary History in Communities of Digital Organisms
Source: PLoS One. 2012 May 31;7(5):e37233. doi: 10.1371/journal.pone.0037233 (PMC3365035; doi:10.1371/journal.pone.0037233)

**SUPPLEMENTARY DATA S1—GENERAL INTRODUCTION TO AVIDA AND CROSS-FEEDING RELATIONSHIPS**

**I. GENERAL EXECUTION IN AVIDA.**

Avida maintains and monitors populations of digital organisms, which are short, self-replicating computer programs written in an assembly-type language. (Any program that can be written with conventional, commercial computer assembly languages can also be written with this language.) The organisms execute the programs encoded by their genomes, including commands that allow them to copy the instructions in their genomes and divide to produce a daughter organism. The copy instruction duplicates a single instruction from parent to daughter. During the duplication process, the instruction being copied has a probability of being miscopied and changed to a different instruction from the one in the parent organism’s genome. Mutations from one instruction to any other in the instruction set are equally likely. In addition, there is a probability that, on cell division, a random instruction will be inserted into or deleted from the daughter cell. These kinds of genomic mutations can indirectly affect the phenotype of a digital organism, including its ability to self-replicate or perform other computational functions. Mutations can also be neutral in their phenotypic effects. Thus, there is a genetic basis for adaptation and speciation (insofar as that can be defined for asexual organisms, not just in Avida but for real biological asexuals). Because the range of variation possible in Avida organisms is indefinite (certainly astronomically large), the system is capable of open-ended evolution. The organisms in this study are completely asexual, but the focus here is on radiation into groupings that are functionally and phylogenetically distinct, followed by loss and regeneration of those groupings. These processes are relevant to both sexual organisms and asexual organisms.

Each digital organism occupies a cell in a rectangular memory lattice, the size of which sets the maximum population size. When an organism divides, the daughter organism is placed in one of the eight immediately neighboring cells, killing any previous occupant of that cell. Organisms that replicate faster have a selective advantage and can more quickly overwrite slower replicators. In Avida, organisms can accelerate the execution of their genomic instructions if they evolve the ability to perform certain logic functions (Lenski et al. 2003). All organisms receive a basal number of CPU cycles (the organisms’ “energy”), which enables their programs to run. If an organism can perform one or more logic functions, it metabolizes a corresponding resource into additional CPU cycles that accelerate the execution of its genome. This creates differences in the rates at which organisms execute their genomes, depending on which functions they perform and which resources can be accessed for additional energy. The values of the energy rewards an organism receives for performing certain computations and consuming certain resources can be set by the experimenter.

**II. LOGIC OPERATIONS IN AVIDA**

When a digital organism in Avida performs one of nine basic logic operations on one or two random 32-bit strings and then outputs the bitwise-correct result, it obtains additional energy (in the form of additional CPU cycles) that accelerates the execution of the instructions in its genome. The energy obtained for correctly performing these logic functions is added to basal energy (which is the same for all organisms) and determines the relative speed with which each organism can replicate its genome.

The logic rules for these nine basic operations are as follows:

Inputs Logic operation

A B NOT NAND AND ORN OR ANDN NOR XOR EQU

0 0 1 1 0 1 0 0 1 0 1

0 1 1 1 0 0 1 0 0 1 0

1 0 0 1 0 1 1 1 0 1 0

1 1 0 0 1 1 1 0 0 0 1

For example, if bit A = 0 and bit B = 0, then (A EQU B) = 1. These rules are defined on single-bit inputs.

In order for an organism to be rewarded for performing an operation, it must perform that operation correctly on all 32 bits of the input strings. The NOT operation is performed with only one input string. Consider an organism that obtains the following two inputs and then executes a series of instructions that results in the following output string:

Input A: 010101011100000000111010101100

Input B: 100001101010001111010110011110

Output: 001011001001110000010011001101

The organism would receive the energy reward for performing the EQU operation because it correctly calculated the EQU function for all 32 pairs of the corresponding bits for inputs A and B and output the correct result.

In spite of the fact that Avida communities are instantiations of Darwinian evolution, metabolism of resources is simulated in Avida because many of its features either are abstract simplifications of or do not correspond directly to real-world biochemical metabolisms. The internal movement and manipulation of numbers between various components of the virtual CPUs (stacks, registers, etc.), however, instantiate what is arguably the computational analogue of metabolism: the numbers are analogous to substrates and the CPU components to enzymes that modify those substrates without themselves being altered. In the cross-feeding trophic network used for these experiments (see fig. 1), successful execution of a logic operation results in consumption of the resource linked to that operation, receipt of the energetic reward (additional CPU cycles) associated with it, and production of a designated resource (possibly more than one) mapped to another operation on the next higher trophic level. In practice, the quantities of produced resources (which are available to all organisms anywhere in the population) are incremented by the Avida software on successful completion of the resource-producing operation and are decremented by the activity of any organisms able to consume those resources (by performing the appropriate operations).

**III. DETAILS OF ENVIRONMENTAL CONFIGURATION**

All experiments are performed using Avida, version 2.1, executed on a Beowulf cluster made up of Intel Pentium III and IV processors and AMD Athlon processors.

Each replicate is seeded with a single copy of a handwritten “default” Avida ancestor, of genome length 50 instructions. This ancestor has no inherent functionality beyond the ability to self-replicate and thus no history of previous functionality. The initial conditions for each replicate differ only in the value of the seed supplied to Avida’s random-number generator. All organisms are asexual; recombination does not occur. All replicates are performed with populations of maximum size N = 3,600. An organism can die either when it is replaced by newborn individuals or when its total instructions executed exceed 20 times its genome length (this prevents nonreproductive organisms from persisting indefinitely). The copy mutation rate is set at 0.005 per instruction copied; insertion/deletion mutations occur at a rate of 0.05 per division. Genome size is not constrained in these experiments: though organisms may allocate only as much memory for offspring as their own genome size, size

change can occur in small increments through insertion/deletion mutations applied on division, avoiding large jumps in genome size change (Misevic et al. 2006). Newborn organisms replace randomly chosen organisms in their immediate eight-cell neighborhood, giving rise to spatially structured populations. Logic operations that are rewarded are those described by Lenski et al. (2003), with rewards scaled as a function of computational difficulty as described by Cooper and Ofria (2002) and Chow et al. (2004). Resources are globally available to all organisms, with no spatial structure. Only resources corresponding to the functions NOT and NAND are provided exogenously, at inflow rates of 200 units/update for each resource, following Cooper and Ofria (2002). There is an additional “infinite” resource supplied to all organisms, which supplies basal energy and is necessary for any execution to occur at all. All other resources arise as by-products of function execution, according to the stoichiometric scheme shown in Supplementary Figure S1. Organisms can obtain a maximum of 25 units of resource or 0.25% of the total concentration (whichever is smaller) per completed computation; the latter condition prevents negative values arising from finite time steps.

**IV. CROSS-FEEDING INTERACTIONS.**

The virtual environment in our experiments has depletable resources, meaning an organism’s access to a given resource is reduced as the resource is consumed by competitors (Cooper and Ofria 2002; Chow et al. 2004). While the experimenter must specify in advance how functions performed by the digital organisms are mapped to resources, the particular organisms that use those resources—as well as the manner in which they do so—may evolve freely. Biotic interactions are simple and facilitative: the digital organisms consume resources and generate byproducts that can themselves serve as resources for other individuals, permitting construction of cross-feeding, codependent environments with trophic structure. Therefore, the disappearance of organisms producing certain resources can also result in the extinction of other organisms dependent on those resources. A schematic of the interactive network used is shown in Supplementary Figure S1. For example, the simple logic function NOT is linked to three higher-level functions (AND, ORN, and OR), and must be performed by an organism three times—each time consuming a unit of the resource mapped to NOT—in order to produce one unit of each resource mapped to those higher-level functions (a 3:1:1:1 conversion). Consumption on higher trophic levels works similarly. This arrangement emulates a directed-energy-transfer system where efficiency decreases between levels (Lindeman 1942) and produces a trophic pyramid where most of the total functionality of the community (and thus produced energy) is at the bottom, decreasing progressively at higher levels. There are no poisons, and “downward” movement of resources from higher levels to lower levels is not implemented. Usually, the digital organisms evolve the ability to perform several kinds of logic function per organism (Cooper and Ofria 2002; Chow et al. 2004), so a given organism may sometimes use its own by-products as additional resources.

We emphasize that this arrangement is not a true food web because true predation (direct consumption of one organism by another) is not implemented. Rather, the cross-feeding relationships that arise among the digital organisms more closely resemble the coexisting bacterial ecotypes reported by Rozen et al. (2005), where a later-evolving type develops the ability to consume metabolites produced by an earlier-evolved type, thus enabling mutual coexistence. Further, the arrangement and stoichiometry shown in Supplementary Figure S1 are user-configured, not inherent to the system. Alternate environment setups that either use direct 1:1:1:1 conversions or have all resources provided at equal inflow rates without any ecological interactions are unsatisfactory, in that they produce unrealistic, inverted trophic pyramid structures where the middle and higher levels have more total functionality.

**SUPPLEMENTARY REFERENCES NOT CITED IN MAIN TEXT**

Misevic D, Ofria C, Lenski RE (2006) Sexual reproduction reshapes the genetic architecture of digital organisms. Proc Roy Soc B 273: 457–464.

Lindeman R (1942) The trophic-dynamic aspect of ecology. Ecology 23:399–418.

Rozen DE, Schneider D, Lenski RE (2005) Long-term experimental evolution in *Escherichia coli*. XIII. Phylogenetic history of a balanced polymorphism. J Mol Evol 61: 171–180.

**Figure S1. Schematic of the cascading trophic interactions used in this study (goes with Supplementary Section 1).** Resources are associated with each of the logic functions shown. The reward value for performing the particular function is shown in parentheses next to the function name. A line connecting resources signifies that an organism performing the lower-level function consumes the incoming resource and produces a by-product that is available for any organism that can perform the higher-level function. Only the resources associated with the lowest-level functions NOT and NAND are provided exogenously. Example conversion factors are shown to the right of one of the inflowing resources and on the connection arrows; in this case, three units of the NOT resource are required to produce one unit each of the resources for AND, ORN, and OR. Similarly, three units of the AND resource are required to produce one unit each of the ANDN, NOR, and XOR resources.


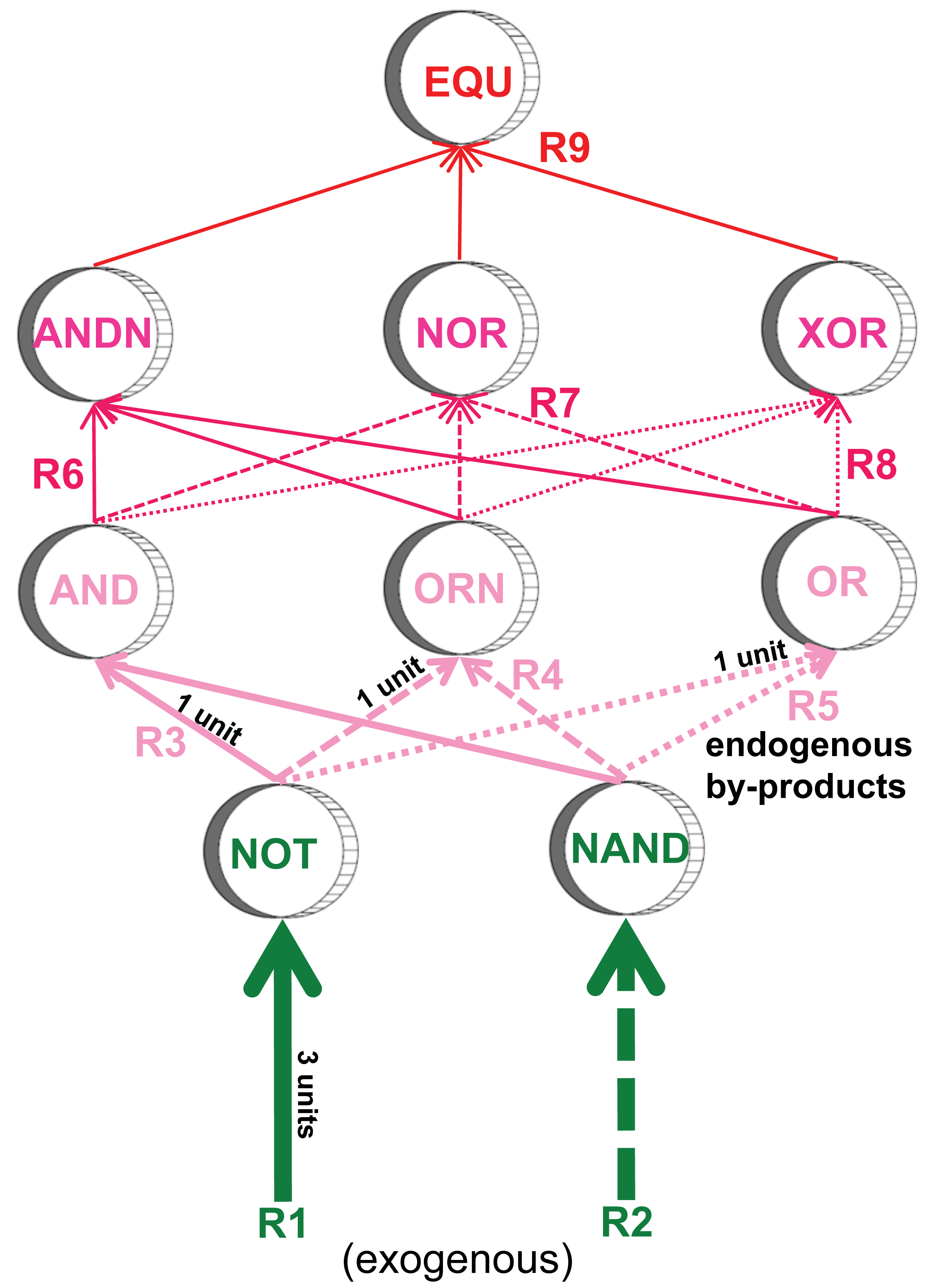

Supplement: Data S1 — General introduction to Avida and cross-feeding relationships. (DOC) [file pone.0037233.s009.doc]
