# Supplementary material for: A Comparison of the Effects of Random and Selective Mass Extinctions on Erosion of Evolutionary History in Communities of Digital Organisms
Source: PLoS One. 2012 May 31;7(5):e37233. doi: 10.1371/journal.pone.0037233 (PMC3365035; doi:10.1371/journal.pone.0037233)

**SUPPLEMENTARY DATA S2—USING LINEAGE-THROUGH-TIME DATA TO ASSESS EROSION OF EVOLUTIONARY HISTORY IN PHYLOGENIES**

One way of localizing the temporal position of loss of history is to plot LTT data taken over several successive time points as histograms. Figure S4 shows an example of LTT data from a single replicate population, plotted for control vs. strong press extinction treatments. In these plots, the x-axis has been truncated at the onset of extinction (100,000 time steps) to focus only on the phylogeny’s pre-extinction history, and the y-axis truncated in order to make the small bars representing deep-branching events easily visible. A great majority of the branching nodes are concentrated near the extinction horizon, with a deficit of branching events particularly between the beginning of the run and the midpoint of pre-extinction evolution (t=40,000-60,000 Avida updates). As the control experiment (Fig. S4a-d) progresses, nodes close to the extinction horizon (those extant at that time of sampling) are much more likely to be lost than nodes representing deeper branching events, although some loss of older nodes does occur (Figure S4c,d, dotted circles). The numbers above the bars are the percentages of nodes from the pre-extinction phylogeny in those temporal bins that are still present in the phylogeny at t=105,000 updates.

The press episode greatly accelerates erosion of history (Figure S4e-h), such that even by the midpoint of the press episode (t=102,500 Avida updates, Figure S4g), the total loss of history in all bins already exceeds that in the control experiment over the entire time corresponding to the press episode. Older nodes remain largely unaffected (at least up to this point), so the phylogeny retains most of the pre-extinction population’s early branching history. By the end of the press episode, only two nodes from the pre-extinction population’s phylogeny remain present in the phylogeny of the end-press population. One of these is the phylogenetic root itself, while the other surviving node has an age between 70,000 and 80,000 updates. All earlier and later nodes from the pre-extinction phylogeny have been lost, leaving a post-extinction tree containing almost no pre-extinction branching.

**Figure S4. Graphical representation of loss of branching history, using lineage-through-time (LTT) data visualized as histograms (goes with Supplementary Data S2).** The x-axis is bins of node ages up to 100,000 updates, the y-axis is number of nodes in the bin. Each bin represents a width of 10,000 updates. Nodes are dated using age in updates. A node whose age falls between time *t* and (*t*+10,000) is placed in the appropriate bin. Since the last bin (95,000 – 100,000 updates) always contains many more nodes than older bins, the y-axis has been truncated to permit visualization of bars in older bins.

Panels a-d) LTT histograms for a representative control experiment at a) immediate pre-extinction, b) 101500 updates, c) 102500 updates, d) 105000 updates. Reduction of bar height in most recent bin cannot be seen due to y-axis truncation.

Panels e-h) LTT histograms for the same absolute time points in the corresponding replicate population during a strong press episode. Panel e) is identical to panel a) above.


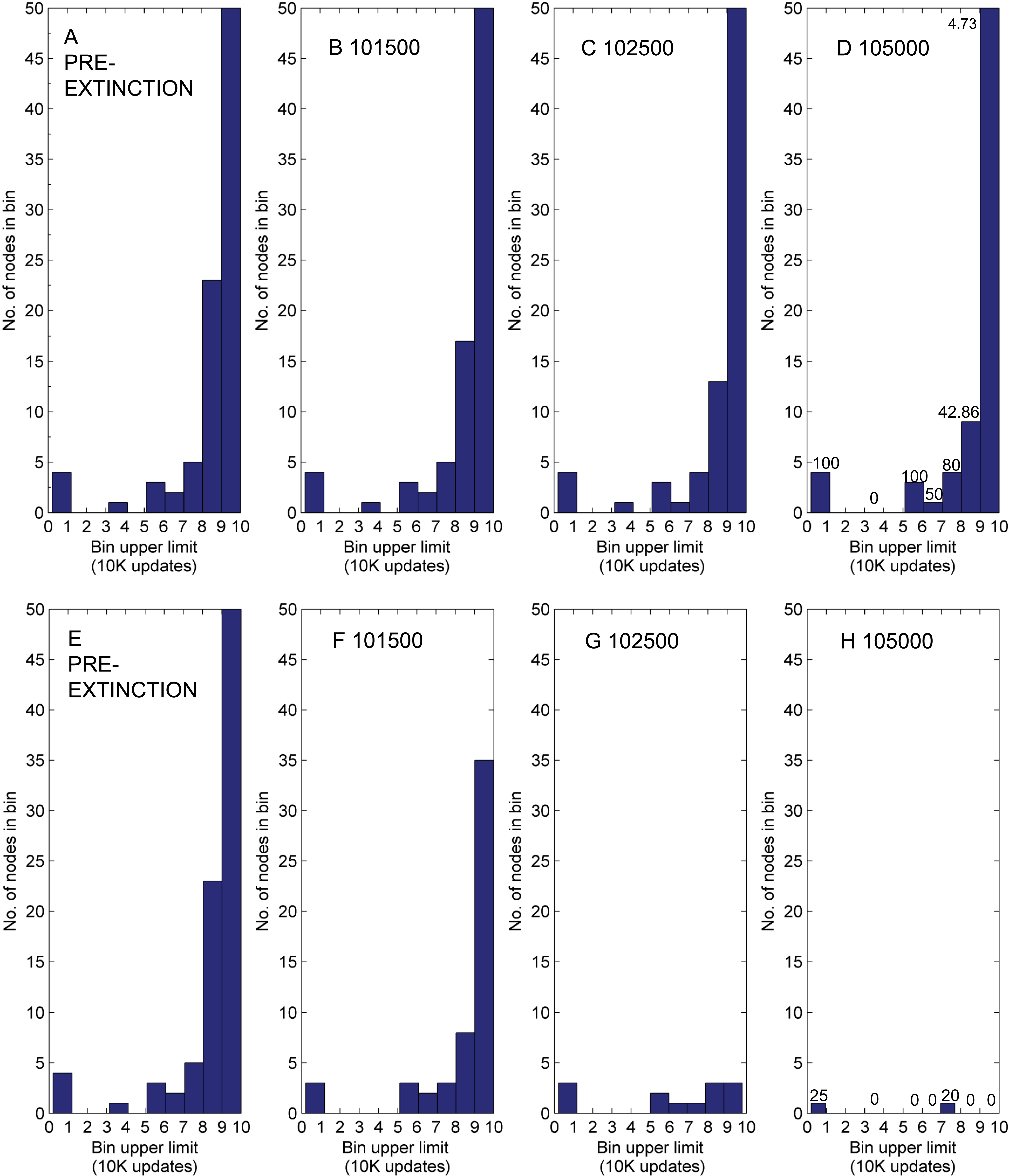

Supplement: Data S2 — Using lineage-through-time data to assess erosion of evolutionary history in phylogenies. (DOC) [file pone.0037233.s010.doc]
