# Supplementary material for: A Comparison of the Effects of Random and Selective Mass Extinctions on Erosion of Evolutionary History in Communities of Digital Organisms
Source: PLoS One. 2012 May 31;7(5):e37233. doi: 10.1371/journal.pone.0037233 (PMC3365035; doi:10.1371/journal.pone.0037233)
Supplement: Data S3 — Definitions of stemminess metrics Pybus-Harvey gamma (PHG) and noncumulative stemminess (NCS). (DOC) [file pone.0037233.s011.doc]

**SUPPLEMENTARY DATA S3—DEFINITIONS OF STEMMINESS METRICS**

**PYBUS-HARVEY GAMMA (PHG) AND NONCUMULATIVE STEMMINESS (NCS)**

1) Pybus-Harvey Gamma (**PHG**). This statistic is measured using lineage-through-time (LTT) data from a molecular (i.e. derived only from taxa extant at the time of sampling) phylogeny. Let g_2_, g_3_, g_4_…g_n_ be the internode distances in a phylogeny with **n** lineages. Then:

PHG assesses where the centre of gravity of a tree lies with respect to the expectation under a pure birth process. Negative values of PHG indicate nodes are concentrated towards the root, indicating the rate of diversification has decreased over time. For calculation of PHG at various time points, the “present” was taken to be the time of sampling.

2) Rohlf et al.’s noncumulative stemminess (**NCS**). This formula is modified from Rohlf et al. [49], and is the one actually implemented in the TreeLoader software used to calculate tree shape statistics.

Where *n* is the number of internal nodes in the tree (excluding the root itself), w*_j🡪i_* is the distance from an internal node *i* to its ancestor node *j*, and h*_j_* is the distance from ancestor node *j* to the root. NCS is an average of the ratios of {distance from internal node to ancestor node}:{ancestor node to root}, taken over all interior nodes excluding the root. NCS values less than 1 indicate long branches from the root before the next branching events; values greater than 1 indicate short branches near the root before the next branching events.
