# Supplementary material for: A Comparison of the Effects of Random and Selective Mass Extinctions on Erosion of Evolutionary History in Communities of Digital Organisms
Source: PLoS One. 2012 May 31;7(5):e37233. doi: 10.1371/journal.pone.0037233 (PMC3365035; doi:10.1371/journal.pone.0037233)

**SUPPLEMENTARY DATA S4—CHANGES IN PYBUS-HARVEY GAMMA**

**LINKED TO CHANGES IN GENOTYPIC TURNOVER**

In the press extinction experiments, PHG values tended to be much more strongly positive at the end of the press episode, a striking finding that cannot be explained by changes in tree size alone. However, only a significantly negative PHG value is informative (as the signal of decreasing rates of diversification). If the value of PHG is either non-significantly negative, or positive, this may mean exponential diversification, or turnover at constant diversity. In order to determine what the increased positive PHG value may be masking, a more direct way of looking at turnover of genotypes is employed.

The inspiration for this analysis comes from paleobiological diversity-through-time studies, which examine changes in origination and extinction of taxa at particular taxonomic levels between stratigraphic bins, thus allowing a direct assessment of turnover. In order to employ such an approach in the genotype-based, high-mutation context of Avida, where most genotypes that exist have rather limited lifespans (a median duration of about 200 Avida updates under the conditions used for these experiments), data on currently living populations must be saved at very frequent time intervals, around every 10 updates. An exemplar strong press extinction experiment was re-run under these conditions, saving information on the currently living population every 10 updates from 5000 updates before the onset of the press episode, during the press episode, and 5000 updates afterwards. The resulting data were analyzed in the following manner:

i) if a particular named genotype is present in a population from time point *x* and also at time *x+t* (where t is an additional time interval, in updates), it persists.

ii) if a particular named genotype is present in a population from time point *x*, but is not present at time *x+t*, it has gone extinct.

iii) if a particular named genotype that is present in a population at time *x+t* was NOT present at time point *x*, it is a new origination.

When plotted over time (Fig S6a), we find that originations and extinctions appear to be fairly balanced against each other prior to the press episode, a sign the community is at or near equilibrium. At the onset of the press episode, the behaviour of the curves suddenly changes, with a series of rapid (but damping) spikes in both origination and extinction as the community adjusts to the low-resource conditions of the press episode. After about 500 updates into the press episode, both originations AND extinctions increase steadily over the duration of the press episode (while persistence declines), although a slight plateau appears to be evident near the end. Since both origination and extinctions increase, we infer that the overall rate of genotypic turnover increases during this time: new genotypes are both being born and going extinct faster than before the press episode. This change is because the population is increasingly dominated by more ecologically streamlined and rapidly-replicating genotypes, resulting in both more individual and genotypic births per unit of absolute time. This period of increased turnover is coincident with the rapid increase in the value of PHG (Fig S6b). When the abiotic environment is restored to its pre-press state, both originations and extinctions relax back towards their pre-press rates, coincident with the sharp decline in the value of PHG. Since this sort of community response occurs across all populations subjected to a press episode, changes in origination and extinction rates, and associated higher or lower genotypic turnover, is a general explanation for the behaviour of PHG in the press extinction experiments. This pattern is robust to temporal degradation of the data (longer time intervals between sampled populations), although the pattern is no longer detectable if the interval greatly exceeds the average lifespan of a genotype.

The same methodology produces a rather different result when applied to a pulse extinction. The example shown here (Fig. S6c) has the same pre-extinction history as the press extinction shown previously, but subjected to a strong pulse extinction (some temporal degradation has been applied to accentuate the differences between the origination and extinction series). Since a large fraction of “biomass” is actually removed from the population, this type of extinction produces a huge spike in genotypic extinction (and a corresponding drop in persistence), without any compensating origination. As the population refills and new diversification occurs, all three measures increase (extinction still occurs during the early phases of the recovery), but there is a short period of time during which originations outpace extinctions. However, extinctions finally catch up to originations, at which point the population is again near equilibrium. The corresponding trend in PHG is shown for this time period in Fig. S6d. There are two strong negative excursions in PHG here, but the first is due to the sudden reduction in tree size (the post-pulse tree is a small sample of the pre-extinction tree). During the recovery, PHG reaches its minimum value (within the limit of sampling resolution used here) at around 102,000 updates, shortly after originations and extinctions have roughly evened out, and becomes positive after that. This result is consistent with the finding that a signal of decreasing rates of diversification can only be detected when a clade first hits equilibrium diversity, or very shortly thereafter [23]. Thus, the differing behaviour of PHG between press and pulse extinctions is rooted in different behaviour of genotypic turnover in the two treatments.

**Figure S6. Differing origination/extinction dynamics of genotypes underlie behaviour of PHG in press vs. pulse extinctions (goes with Supplementary Data S4).** In panels a and c, blue series—originations, red series—extinctions, black series—persistences.

a) Origination/extinction dynamics for a representative Strong Press experiment.

b) Corresponding change in PHG vs. time for panel a).

c) Origination/extinction dynamics for a representative Strong Pulse experiment.

d) Corresponding change in PHG vs. time for panel c).


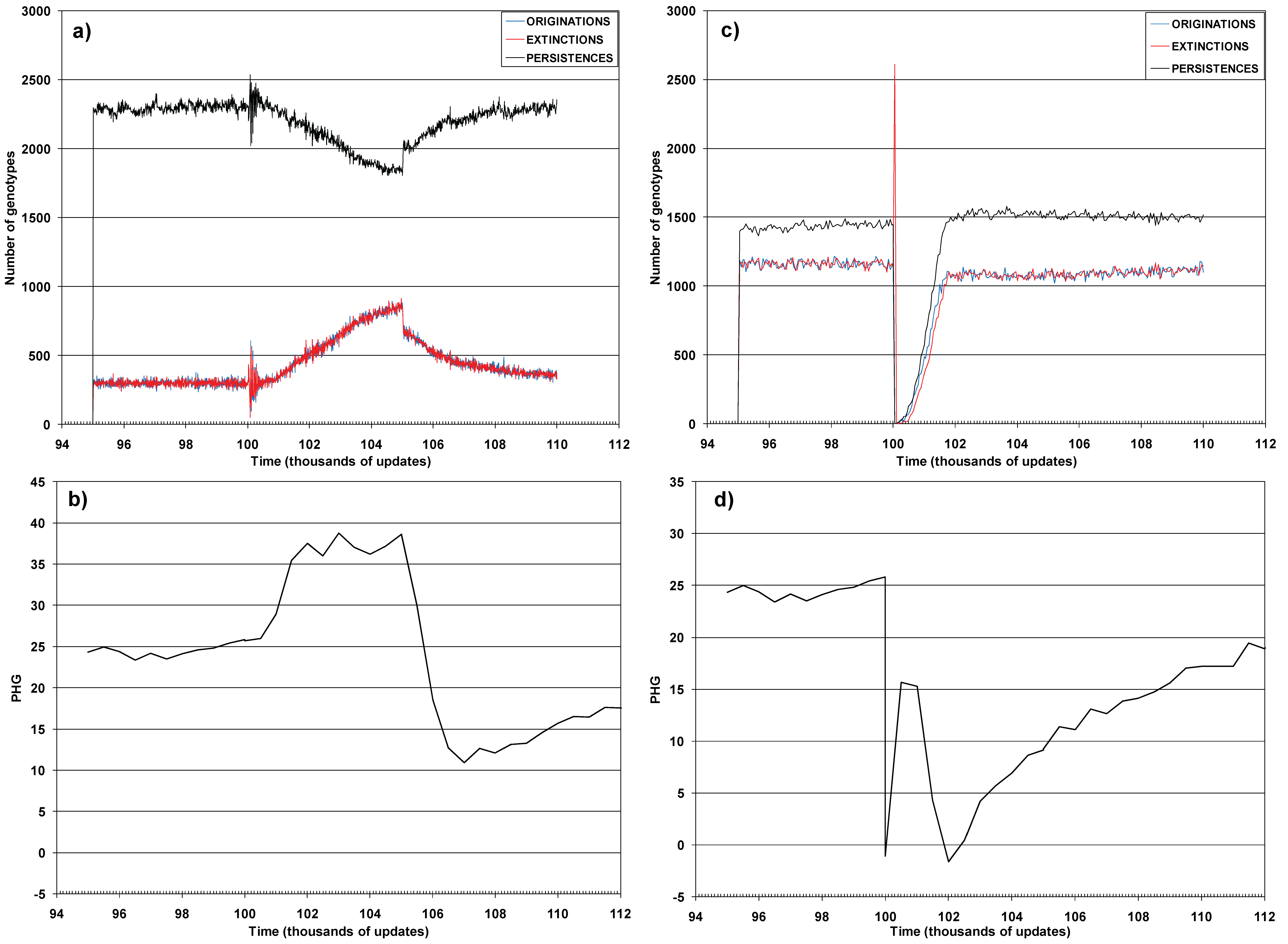

Supplement: Data S4 — Changes in Pybus-Harvey gamma linked to changes in genotypic turnover. (DOC) [file pone.0037233.s012.doc]
